# Identification and Drug Screening of Single Cells from Human Tumors on Semiconductor Chip for Cancer Precision Medicine

**DOI:** 10.1002/advs.202503131

**Published:** 2025-04-24

**Authors:** Wenhao Hui, Ka‐Meng Lei, Yingying Liu, Xinru Huang, Yunlong Zhong, Xiaojun Chen, Mingji Wei, Jie Yan, Ren Shen, Pui‐In Mak, Rui P. Martins, Shuhong Yi, Ping Wang, Yanwei Jia

**Affiliations:** ^1^ State Key Laboratory of Analog and Mixed‐Signal VLSI Institute of Microelectronics University of Macau Taipa 999078 Macau; ^2^ Faculty of Science and Technology University of Macau Taipa 999078 Macau; ^3^ Liver Transplantation Center The Third Affiliated Hospital Sun Yat‐Sen University Guangzhou 510000 China; ^4^ Department of Hepatobiliary Surgery The First Affiliated Hospital of Guangzhou Medical University Guangzhou 510000 China; ^5^ Lingnan Normal University Zhanjiang 524000 China; ^6^ Electrical and Information Engineering Jiangsu University Zhenjiang 212000 China; ^7^ Department of Physics National University of Singapore Singapore 546080 Singapore; ^8^ Mechanobiology Institute National University of Singapore Singapore 546080 Singapore; ^9^ On leave from Instituto Superior Tecnico Universidade de Lisboa Lisboa 1649‐004 Portugal; ^10^ MoE Frontiers Science Center for Precision Oncology University of Macau Taipa 999078 Macau

**Keywords:** cell impedance, drug screening, integrated circuit (IC), precision medicine, single cell

## Abstract

Drug screening of primary tumor cells directly assesses the drug efficacy on specific tumors, promoting personalized cancer treatment. The application of a microfluidic platform has realized drug screening using a limited amount of biopsy samples for cancer precision medicine. However, all the techniques face an inevitable issue of not all the primary tumor cells being cancer cells. Here, a system is introduced that integrates single‐cell identification and drug screening on one semiconductor chip so that both drug efficacy on cancer cells and drug toxicity on noncancerous cells can be obtained simultaneously. An integrated circuit is built on the semiconductor chip for single‐cell electric impedance sensing (IC‐ECIS) of ultra‐weak signals for distinguishing cancer cells from noncancerous cells without affecting cell vitality. Single‐cell identification is validated using breast, lung, and liver cell lines as well as liver cancer specimens from clinical patients. The accuracy on commercial cell lines is ≈80%, and the diagnostic results of tumor tissues are consistent with clinical pathology results. Drug screening is run on the same chip after single cell identification for dual evaluation of drug efficacy and toxicity in both breast cancer models and clinical liver cancer patients. The on‐chip drug screening is confirmed with off‐chip counterpart experiments in breast cell lines. The effectiveness or ineffectiveness of a drug screened on the IC‐ECIS chip demonstrated consistency in the presence or absence of specific mutations in the drug‐related genes determined via exome sequencing of individual liver tumors, validating the method for precision medicine.

## Graphical abstract

The study innovatively integrates single‐cell electric impedance identification and drug screening on a semiconductor chip, addressing tumor heterogeneity limitations. The system achieves label‐free cancer cell recognition while concurrently evaluating drug efficacy on cancer cells and toxicity on noncancerous cells. Clinical validation confirmed alignment with pathology and exome sequencing, advancing precision oncology platforms.

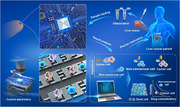

## 1 Introduction

Precision medicine in oncology aims to tailor treatment strategies for individual cancer patients based on the distinctive cellular and molecular traits of their tumors, aiming for optimized therapeutic outcomes.^[^ ^1^, ^2^, ^3^ ^]^ Clinicians analyze genetic mutations or other molecular features of individual tumors to maximize treatment effectiveness.^[^ ^4^, ^5^, ^6^ ^]^ However, predicting outcomes solely based on mutations of specific genes poses challenges due to the intricate drug pathways involving numerous genes.^[^ ^7^, ^8^, ^9^ ^]^ A more direct approach involves screening drugs at the cellular level using primary tumor cells, offering immediate insights into how a specific tumor responds to particular drugs and avoiding uncertainties linked to genetic testing of specific genes.^[^ ^6^, ^10^, ^11^ ^]^ Various innovative platforms have been developed to improve the drug screening process for cancer treatment, utilizing small amounts of primary tumor cells obtained from biopsy samples.^[^ ^12^, ^13^, ^14^ ^]^

Nevertheless, a significant challenge persists: biopsy samples from patients typically comprise a mixture of cancer and noncancerous cells, which can result in imprecise assessments of drug efficacy if all the cells are considered the same. This highlights the crucial requirement for techniques capable of distinguishing cancer cells from noncancerous cells within heterogeneous populations, ensuring precise and effective drug screening.

Currently, two main strategies exist for distinguishing between cancerous and noncancerous cells: chemical and physical methods. Chemical identification of cancer cells involves using specific biomarkers found on the membranes of certain cancer cells. For instance, Qu et al. crafted an electrochemical aptasensor designed to detect cancer cells by targeting nucleolin, a molecule overly expressed on their surfaces, and verified its effectiveness across various cell lines.^[^ ^15^ ^]^ In a separate study, Hao et al. investigated the application of a hemocyanin dye for selective binding to various cellular organelles, allowing differentiation between cell types based on observed differences in average fluorescence intensity.^[^ ^16^ ^]^ However, it is crucial to note that the presence of specific compounds on cell membranes or within cell plasmids can impact protein distribution. These proteins may either facilitate or impede drug entry into cells, potentially inducing misleading results in drug tests. To minimize the influence of surface proteins, researchers have explored the use of physical characteristics to differentiate cancer cells from noncancerous cells. Huang et al.^[^ ^17^ ^]^ and Dong et al.^[^ ^18^ ^]^ utilized an optical stretcher beam to observe cell stretching and deformation, noting the greater flexibility of cancer cells compared to noncancerous cells. However, this method caused irreversible mechanical damage to cells and had low throughput. Importantly, these methods do not allow for cell retrieval post‐identification, which is crucial for subsequent drug screening applications.

In recent years, the technology of electric cell‐substrate impedance sensing (ECIS) has emerged as a highly convenient and safe method for the identification of cells and the assessment of their viability. Its benefits include its non‐invasive, label‐free nature and its capability for real‐time analysis.^[^ ^19^, ^20^ ^]^ The response of cells to a low voltage alternating current reveals two primary types of information: capacitive and resistive properties. Cell morphology is usually considered as a parameter that affects capacitance changes. Conversely, the resistive component of the impedance is largely a function of cleft or adhesion resistance, followed by cytosolic resistivity.^[^ ^21^, ^22^ ^]^ The seminal works by Giaever and Keese from the 1980s and 1990s laid the groundwork for ECIS by introducing impedance monitoring of cell behavior in tissue culture and provided essential insights into the signals generated by cell‐substrate interactions.^[^ ^23^, ^24^, ^25^ ^]^ To advance single‐cell analysis through impedance sensing, Gelsinger et al. developed a micromachined impedance spectroscopy flow cytometer capable of multi‐frequency measurements (100 kHz to 15 MHz) to classify cells at the single‐cell level.^[^ ^26^ ^]^ Zhao et al. explored single‐cell electrical phenotyping using microfluidic platforms to classify tumor cells based on specific membrane capacitance and cytoplasm conductivity.^[^ ^27^ ^]^ These innovations led to the creation of microelectrodes tailored for cell‐sized impedance sensing. While this method enhanced accuracy, its effectiveness was hampered by one cell per second process, making it unsuitable for large clinical samples. To overcome this issue, Joshi et al. integrated nanoparticle‐printed with machine learning algorithms to streamline the capture of single cells for impedance measurements and improve throughput of over 1000 cells per second.^[^ ^28^ ^]^ This system overcame the bottlenecks of low throughput and complex data analysis seen in earlier methods. Despite these advancements, a common challenge persisted: the extended wires in the sensing circuit introduced significant background impedance and noise. This interference obscured the subtle signal variations crucial for distinguishing between different types of single cells, posing a barrier to precise identification and subsequent applications in drug screening.

Integrated circuit (IC) technology is a promising method for detecting weak bioelectric signals by integrating complex computing circuits and wiring onto complementary metal‐oxide‐semiconductor (CMOS) chips, thus reducing signal attenuation and distortion during transmission.^[^ ^29^ ^]^ In the early stages of bio‐CMOS research, Derek et al. developed a portable cell sensing system using CMOS and polydimethylsiloxane to monitor and record the action potentials of HL‐1 cardiomyocytes.^[^ ^30^ ^]^ To enhance detection throughput, Jung et al. designed a high‐throughput multimodal cell sensor and stimulator array chip, featuring 21952 pixels and 1568 parallel reading channels, enabling high‐resolution multimodal detection.^[^ ^31^ ^]^ In the field of impedance sensing, Chitale et al. developed a custom semiconductor 96‐microplate device, demonstrating its ability to measure a wide range of cell types using impedance measurement techniques and accurately distinguish between an array of biological responses to compounds.^[^ ^32^ ^]^ Electrode arrays with a resolution of 25 µm are suitable for relatively stable and uniform sizes of commercial cell lines. However, clinical tumor cells have a larger range of sizes and cannot fit into this resolution, which reduces the utilization rate of precious primary tumor cells. In general, while CMOS technology is advanced in cell signal detection, no existing chip integrates both cell identification and drug screening. More importantly, there is a lack of exploration of introducing IC into clinical environments and in vitro diagnosis.

**Figure 1:**
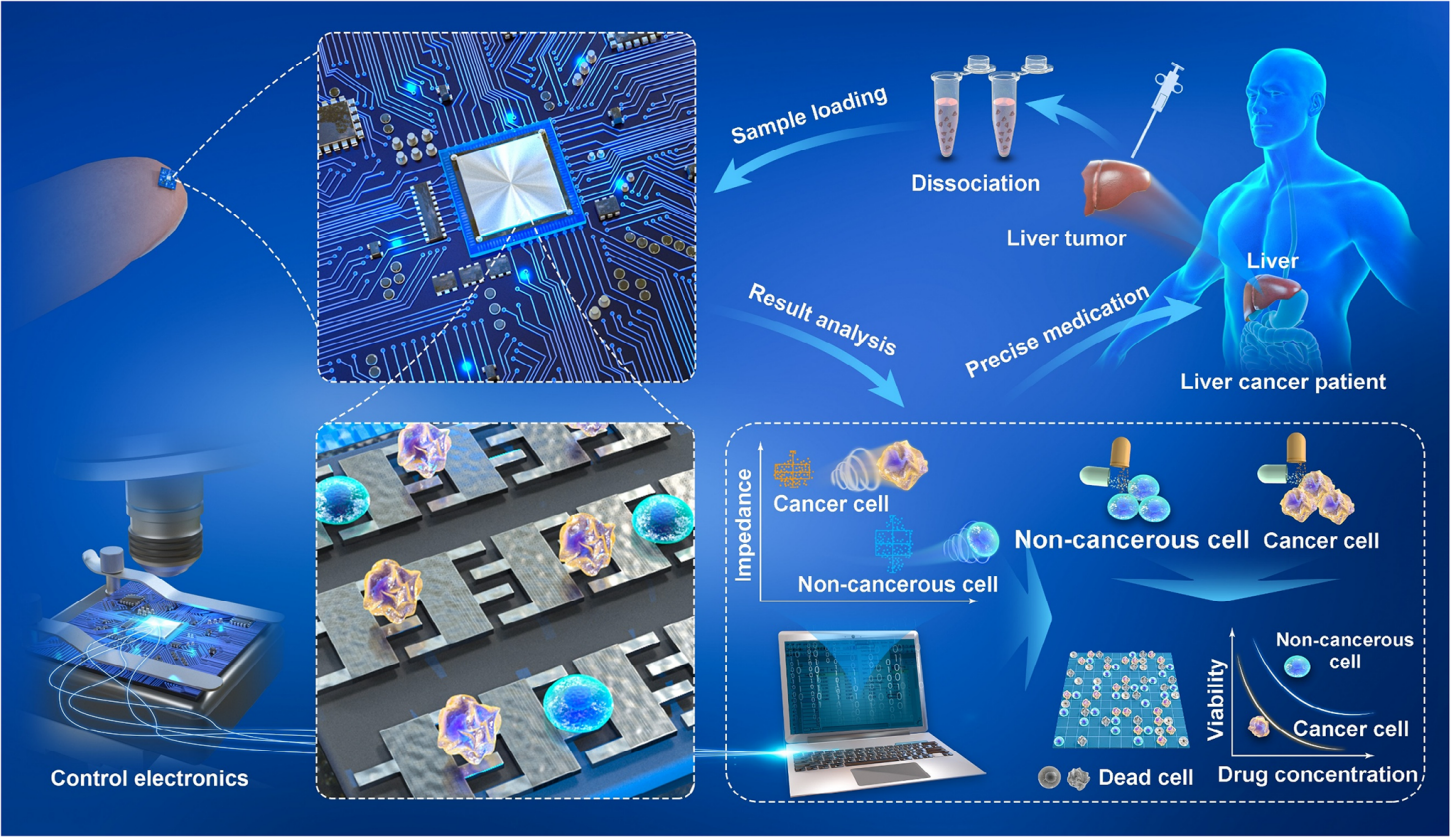
Schematic diagram of integrated circuit (IC) based single‐cell electric cell‐substrate impedance sensing (IC‐ECIS) platform for single‐cell identification and drug screening.

In this study, an IC‐based platform for single‐cell electric cell‐substrate impedance sensing (IC‐ECIS) is introduced, aimed at accurately identifying individual cells and evaluating drug effects. **Figure** **1** displays the comprehensive workflow, from the IC chip design to cell differentiation and its utilization in precision medicine for drug screening. The IC‐ECIS chip represents an innovative integration of an impedance sensing circuit with hundreds of microelectrodes on a semiconductor chip, facilitating the distinction between cancerous and non‐cancerous cells at single‐cell resolution. By situating the amplifier circuit directly below the cell‐sized sensing electrode, the signal‐to‐noise ratio is significantly enhanced, primarily through the reduction of the distance between the signal source and signal sensing. Experiments conducted using the IC‐ECIS chip successfully differentiated commercially available breast, lung, and liver cancer cells from their non‐cancerous counterparts, displaying distinct impedance signatures at the single‐cell level. Additionally, tests with 12 primary liver cancer cells from clinical samples were performed using the IC‐ECIS platform for both cell identification and drug screening. This dual functionality not only confirmed the efficacy of certain drugs against cancer cells but also highlighted their toxicity to non‐cancerous cells. The consistency of cell identification results with pathological diagnoses from hospitals and the confirmation of drug efficacy outcomes through exome genetic sequencing attest to the reliability of the approach. Compared to traditional drug screening methods for primary tumor cells, this approach of first distinguishing cell types before screening improves accuracy. The application of this platform in drug screening could significantly advance cancer precision medicine, enabling the use of limited biopsy samples with rapid processing times.

## 2 Results and Discussion

### 2.1 IC‐Based Single‐Cell Electric Cell‐Substrate Impedance Sensing (IC‐ECIS) Platform

**Figure 2:**
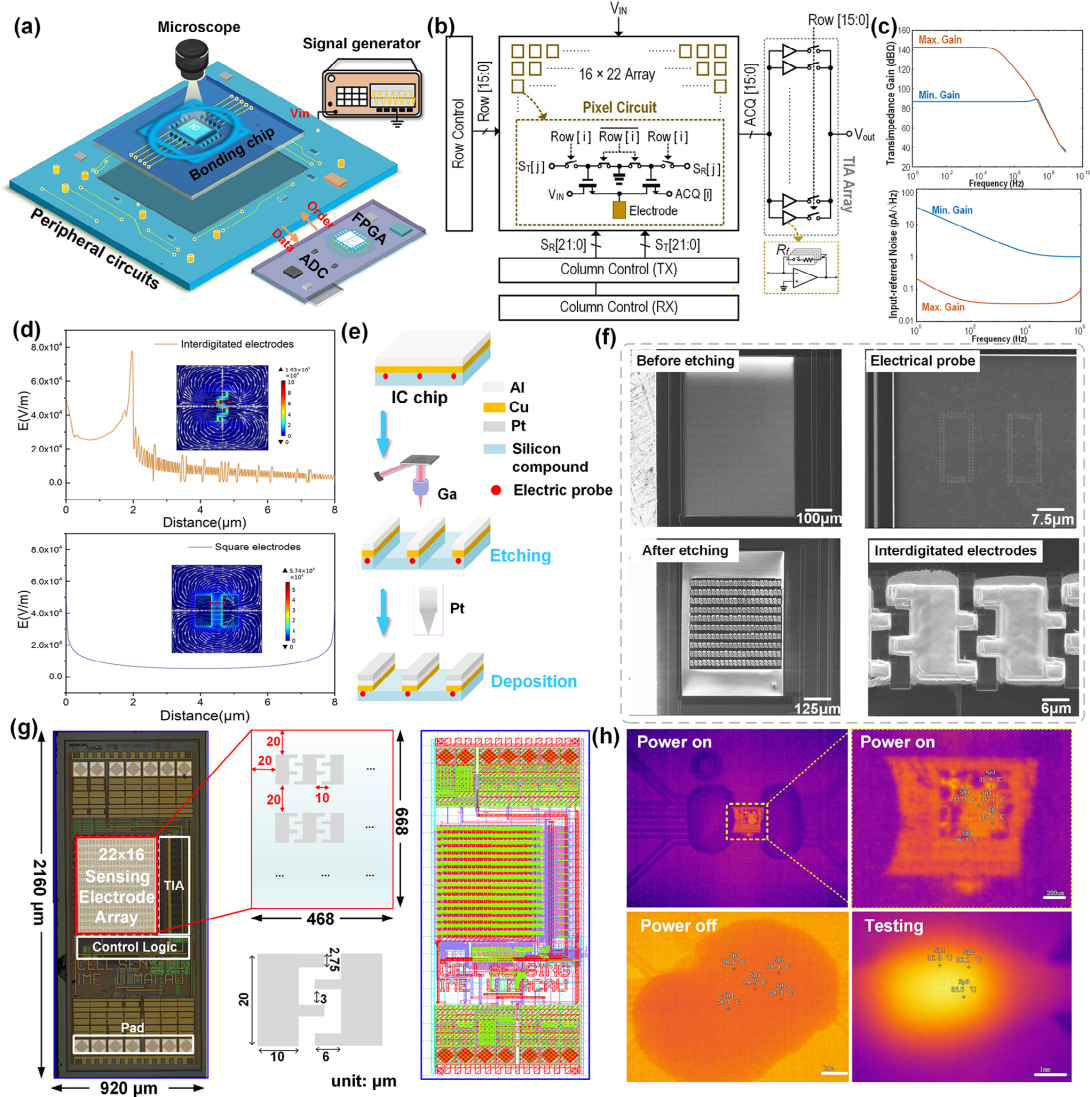
Set up of single‐cell impedance sensing platform and IC chips. a) Single‐cell impedance measurement platform. b) The internal circuitry of the IC chip. c) Simulation results of chip noise and gain. d) Electric field distribution of interdigitated electrodes and square electrodes. e) Focused ion beam (FIB) for electrode fabrication. f) Scanning electron microscopy images before and post fabrication of FIB. g) Photo of IC chip fabricated by AMS 0.35‐µm CMOS technology. h) Thermal distribution of the chip during power‐on, power‐off, and testing.

For single‐cell electrical impedance sensing, a microelectrode array consisting of hundreds of electrodes is required to capture signals from individual cells. **Figure** **2**a illustrates the schematics of the IC‐ECIS platform layout. The sensing IC chip, featuring impedance detection circuits compatible with custom‐designed surface electrodes, is interfaced with peripheral circuitry via gold wires to facilitate communication and data transfer. Upon the deposition of the cell solution onto the chip, the precise locations of individual cells are identified under a microscope. A field‐programmable gate array manages control and data processing, dispatching digital signals to the chip to specifically select electrode rows and columns for measurement. This gate array also retrieves the digitized signal from the IC, converting it into impedance readings. A signal generator supplies the input signal to the chip, activating the designated electrodes. Further details on the IC chips and the experimental setup are provided in the Supporting Information.

In the design of the sensing IC, a transimpedance amplifier^[^ ^33^, ^34^ ^]^ is employed, adept at measuring and amplifying the impedance between adjacent surface electrodes. This design is particularly suited for detecting faint electrical signals from biological entities, such as single cells and microorganisms, by reducing the background impedance typically introduced by long wires. The single‐cell impedance can be calculated using the formula:

(1) $$\left|Z\right|=\left|\frac{-R_{f}V_{OUT}}{V_{IN}}\right|$$

where *V_IN_* and *V_OUT_* represent the input voltage and output voltage of the amplifier, respectively, *R_f_* is the feedback resistor inside the IC chip. A MATLAB program has been developed to perform the automatic batch calculation of equivalent impedance by detecting the input and output voltages between all adjacent surface electrode pairs.

Each electrode pair is independently activated to minimize the electromagnetic interference typically associated with energizing large arrays of electrodes simultaneously. Figure 2b displays the internal circuitry of the IC chip in a schematic diagram. Upon receiving positional data for the test electrode, the row and column control modules initiate the activation of the designated electrode channel. The signals captured through this process are subsequently forwarded to an array of transimpedance amplifiers, consisting of 16 amplifiers in total, each tasked with processing signals from a row of electrodes. The proximity of the signal collection apparatus to the test subjects significantly reduces background noise, thereby enhancing measurement sensitivity down to the level of individual cells.

We simulated the transimpedance gain and input‐referred noise, as indicated in Figure 2c. For max gain mode, the gain is high and stable at frequencies below 10 MHz, meaning the system can strongly amplify weak signals like small impedance changes in single cells. However, above 10 MHz, the gain decreases, indicating a drop in amplification ability at higher frequencies. This drop‐off comes from parasitic capacitance or limitations in circuit design that affect high‐frequency performance. For the min gain mode, the gain is much lower (≈60 dBΩ) and remains flat across frequencies, which may be useful when only low sensitivity is needed. Regarding the noise, it is consistently low across the frequency range in max gain mode. In the min gain mode, the noise is higher at low frequencies, but it stabilizes and becomes lower as the frequency increases. The results show that the system has relatively stable gain and low noise at the low and medium frequency ends. Details on the TIA compensated are provided in the Supporting Information.

After defining the circuit architecture, the objective was to integrate hundreds of single‐cell‐sized electrodes onto the surface of the IC chip to enable direct contact with cells. Optimization of electrode shape patterns is crucial for the sensitivity of single‐cell impedance measurements. We simulated the electric field distribution for both interdigitated and square electrodes in COMSOL. As shown in Figure 2d, the interdigitated electrodes generate a highly concentrated electric field between the fingers, with rapid decay outside this region. This concentrated field enables them to detect localized changes in cell membranes or intracellular properties more sensitively, making them highly effective at capturing subtle electrical characteristics of cells. Importantly, compared with ordinary square electrodes, the interdigitated electrodes provide a larger contact area and a higher probability of single‐cell capture. We ultimately chose interdigitated electrodes as the array pattern. However, fabricating such a vast array of electrodes on an IC chip without compromising the integrity of the underlying circuitry at such fine scales presents substantial technical challenges. To address these, a focused ion beam (FIB) technique was employed for precise processing of the surface electrodes, as illustrated in Figure 2e. During IC fabrication, a 668 × 468 µm^2^ region was used for the exposed 925 nm thick metal layer, consisting of 95% aluminum and 5% copper, following the tape‐out stage. Gallium ions were used to meticulously sculpt the electrode geometries and remove excess aluminum and copper to create isolated electrodes. Subsequently, a layer of biocompatible metal, platinum, was deposited atop the patterned electrodes to enhance their compatibility with cellular adhesion. Figure 2f displays scanning electron microscopy images of the electrode array, both pre‐ and post‐fabrication.

Figure 2g presents a microscopy image of the IC‐ECIS chip, showing precisely patterned surface electrodes. For proof‐of‐concept, the IC chip utilized in this study measured 0.92 mm × 2.16 mm and incorporated a grid of 22 × 16 = 352 surface microelectrodes with the necessary input/output pads. The 10 µm × 20 µm electrodes, separated by a 10 µm gap, were designed to be interdigital with the closest distance of 3 µm to maximize the single‐cell capture rate. To address potential issues of electrical discontinuity beneath the electrodes due to fabrication variations from chip to chip and misalignment during post‐processing, the design included multiple probes beneath each electrode. These were strategically integrated within the IC circuitry to form two looping arrays, ensuring consistent and reliable functionality. Considering the impact of temperature on cell viability, we used a micro thermal imager to observe the thermal distribution of the chip during operation, as indicated in Figure 2h. When the chip was in a working state, the temperature range from 35 to 37 °C at various spots on the chip surface. It remained well within acceptable limits for biological and electronic functionality.

### 2.2 Physical Theory of Impedance for Cell Discrimination

**Figure 3:**
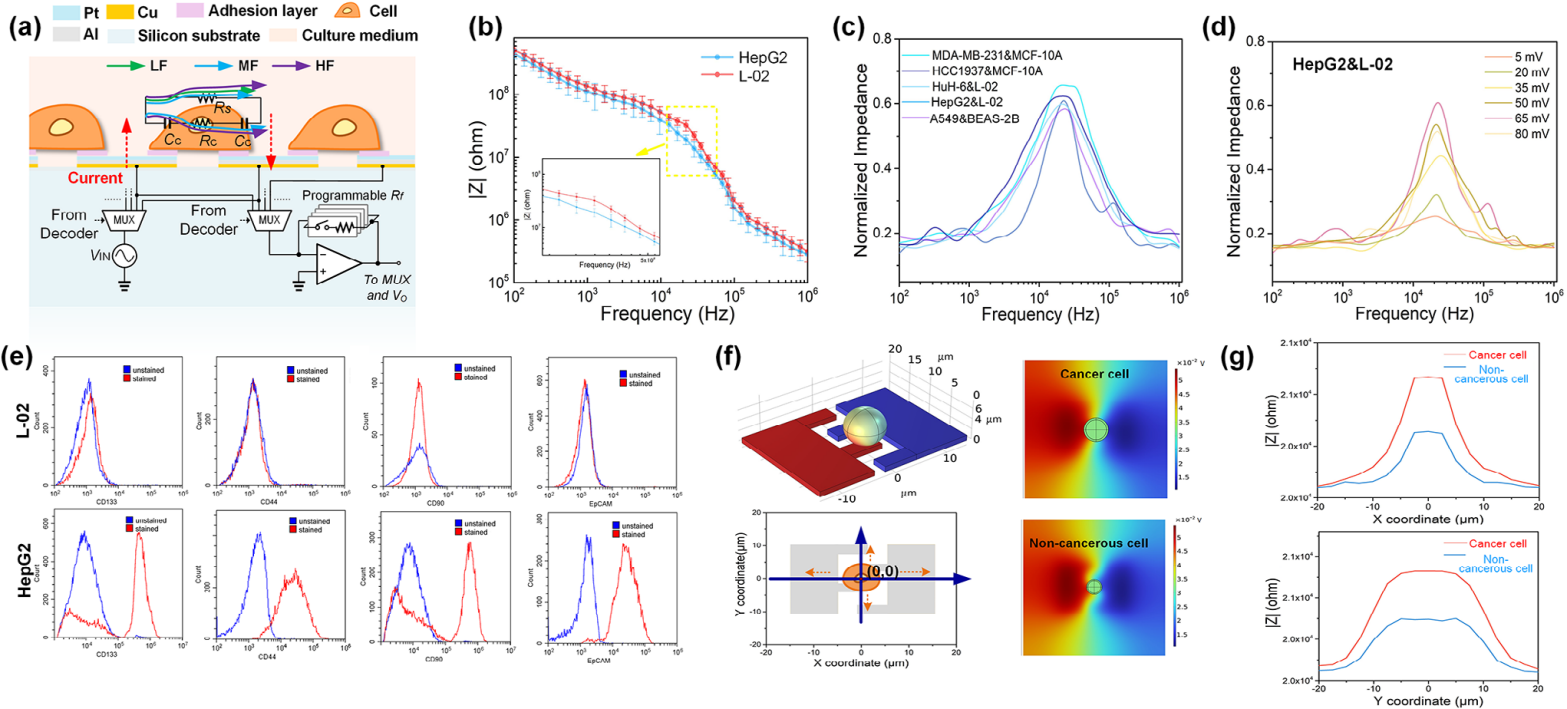
Principle of single‐cell impedance sensing and selection of optimal measurement conditions. a) Cross‐sectional diagram of single cell impedance measurement. b) Sweep frequency diagram of L‐02 and HepG2 cell lines at 100 Hz–1 MHz. Error bars represent standard deviation, *n* = 50 independent cell replicates. c) Normalized impedance of different types of cancer and noncancerous cells. d) Normalized impedance at excitation signal amplitude of 5–80 mV. e) Flow cytometry analysis of L‐02 and HepG2 cells. f) Computational model and single cell movement in CMOSOL. g) Impedance changes as cells move along the X‐axis and Y‐axis.

Before conducting large‐scale single‐cell impedance measurements, we need to understand the physical theory of the IC‐ECIS chip to discriminate cancer and noncancerous cells based on their impedance. **Figure** **3**a presents a cross‐sectional diagram of single cell impedance measurement. The pathway for the current begins at the signal source, traverses through the sensing electrode, the adhesion protein layer, the single cell situated between adjacent electrodes, the culture medium, the opposite electrode, and finally returns to the amplifier circuit. The impedance of a single cell is complex, encompassing its real and imaginary components that denote the resistance to electric current and capacitive reactance to the electric field, respectively. The magnitude of impedance offers a thorough perspective on the cellular reaction to alternating current and serves as a valuable measure for describing the impedance traits of cells at different frequencies. This attribute is particularly beneficial for applications in cell classification, health status assessment, and further classification tasks due to its capacity to provide detailed insights into cellular behavior.^[^ ^35^, ^36^ ^]^

It is important to note that appropriate frequency can improve the sensitivity of single‐cell detection while an unsuitable frequency can lead to unreliable results due to electrode polarization effects and electrical noise.^[^ ^37^ ^]^ The optimization of the electrical signal for impedance measurements was established based on the swept image presented in Figure 3b. We performed a frequency sweep on human liver cells L‐02 and human liver cancer cells HepG2 and found that the impedance magnitude difference between the two cells was the largest when the frequency was 20 kHz. To investigate whether the optimized frequency of 20 kHz on the liver cancer‐noncancerous pair also works the best for other biological models, we applied it to different pairs of breast cancer, liver cancer and lung cancer. Considering that our goal is to distinguish noncancerous cells from cancer cells, and the cell identification effect is the criterion for the most sensitive frequency, we conducted frequency sweeps and normalization on the noncancerous cell lines and their corresponding cancer cell lines. The impedance normalization formula is as follows:

(2) $$NI=\frac{|Z_{C\;}|-\left|Z_{N}\right|}{\left|Z_{N}\right|}\;$$

The commercial breast cancer cell lines (MDA‐MB‐231, HCC1937), liver cancer cell lines (HuH‐6, HepG2), and lung cancer cell line (A549) were tested alongside their respective noncancerous cell lines (MCF‐10A, L‐02, and BEAS‐2B) on the chip. As shown in Figure 3c, the most sensitive frequencies for distinguishing between MDA‐MB‐231 and MCF‐10A, HCC1937 and MCF‐10A, A549 and BEAS‐2B, and HuH‐6 and L02 fall within a specific range. For example, the most sensitive frequencies discriminating MDA‐MB‐231 and MCF‐10A were between 15 and 30 kHz, while the most sensitive frequencies for HCC1937 and MCF‐10A were between 10 and 25 kHz. However, 20 kHz was always within this sensitive frequency range among these models, indicating that it can be used as the test frequency in this study.

Signal amplitude is another key parameter for ultra‐sensitive impedance detection. A lower voltage amplitude could result in a low signal‐to‐noise ratio, while a higher voltage amplitude might cause electroporation or damage to the cells, altering their electrical properties and potentially distorting the results.^[^ ^38^, ^39^ ^]^ To find the most optimized voltage amplitude, we conducted frequency sweeps ranging from 5 to 80 mV. According to the normalized impedance results in Figure 3d, we found that the resolution between the two cell types was optimal at 65 mV. To better understand the physical meaning behind the impedance difference, we extracted the actual impedance information to build the Nyquist plot and the equivalent circuit model, details are shown in the Supporting Information. There are notable differences in adhesion proteins between cancer and noncancerous cells. As indicated in Figure 3e, flow cytometry results show that HepG2 cells have increased levels of CD133, CD44, CD90, and EpCAM proteins. CD133 is a transmembrane glycoprotein that regulates adhesion by mediating interactions between the cytoskeleton and extracellular matrix (ECM). CD44 and CD90 are also involved in cell‐ECM interactions, playing key roles in adhesion, migration, invasion, and signal transduction. EpCAM, a cell surface adhesion molecule, enhances adhesion and regulates cell proliferation, migration, and differentiation via signal transduction pathways. When cells adhere to the electrode surface via these proteins, charge transfer becomes more challenging as it must pass through a larger extracellular adhesion protein, leading to an increase in charge transfer resistance.^[^ ^40^, ^41^ ^]^ Additionally, cancer cells exhibit higher double‐layer capacitance due to their complex membrane structure and greater number of adhesion proteins.

Besides, as cells transit into a cancerous state, they undergo significant changes affecting their physical and chemical characteristics. These alterations include adjustments in cell membrane permeability, transmembrane potential, surface charge, and ion concentration. Notably, cancerous cells exhibit lower levels of sodium (Na^+^) and chloride (Cl^−^) ions inside the cell compared to noncancerous cells.^[^ ^42^ ^]^ This discrepancy results in decreased electrical conductivity and increased impedance when cancerous cells are exposed to an electric field. Additionally, cancer cells tend to release higher quantities of lactate ions, which can cross the cytoplasmic membrane, leading to a substantial buildup of negative charges on the cell membrane surface. When subjected to an electric field, this accumulation of negative charge can generate a current in the opposite direction, thereby enhancing impedance.

The ideal circuit model offers a comprehensive explanation of the electrical interactions between cells, electrodes, and their environment. However, a common challenge arises from the random placement of cells on the electrode array. To address this issue and enable detailed observation of impedance variations among cells at different locations and their unique electrical responses under an electric field, we employed the double‐sphere equivalent model for our COMSOL simulations, depicted in Figure 3f. This model ensures precise alignment of electrode sizes with those on the actual chip surface.

The finite element simulations followed Maxwell's mixture theory, the detailed parameters and formulas can be found in Supporting Information. An alternating current signal with an amplitude of 65 mV and a frequency of 20 kHz was applied to represent cancerous cells modeled as equivalent dielectric spheres. The position of the cell model is adjusted to simulate random adherence to the electrode. As shown in Figure 3g, the impedance of a single cell changes as it moves along the X‐ and Y‐axes. Specifically, at positions less than 3.5 µm from the origin on the X‐axis, the impedance remains relatively consistent. Similarly, stable impedance occurs at a distance of 10 µm from the origin along the Y‐axis. However, as cells move farther from the origin, there is a gradual decrease in impedance. This observation, considering electrode size, suggests that accurate impedance measurements are achievable when cells, whether cancer or noncancerous, are positioned between interdigitated electrodes and simultaneously contact both. Although this simple model may not represent the real situation, it can be used to explain that some misclassifications are likely to be from differences in cell locations.

### 2.3 Discrimination of Commercialized Cancer/Noncancerous Cells

To assess the capability of the IC‐ECIS chip in discriminating single cells, commercial breast, liver, and lung cancer cell lines and their respective noncancerous counterparts were tested alongside on the chip.

**Figure 4:**
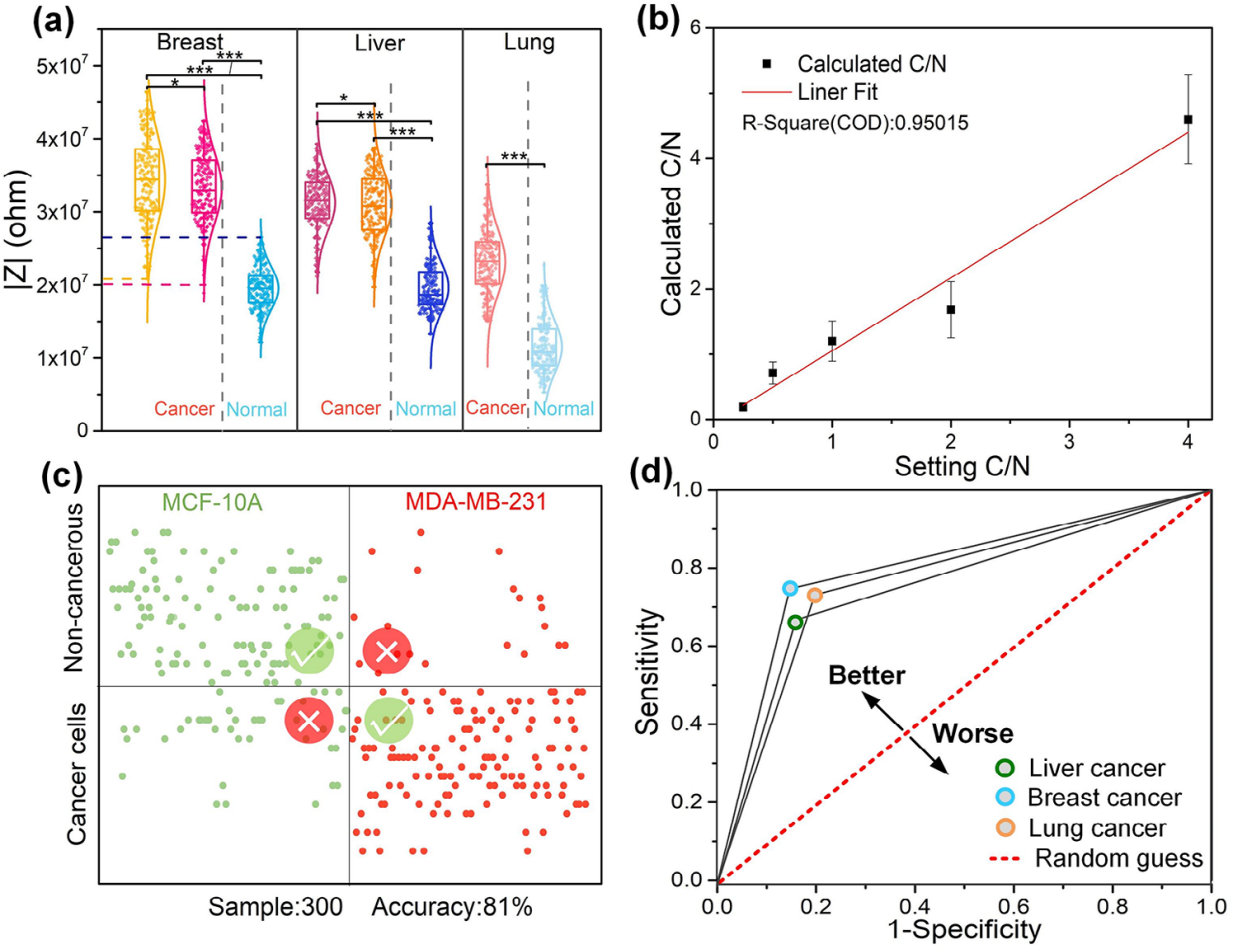
Validation of the single‐cell distinguishing method based on impedance. a) Impedance of human liver cell lines (L‐02) and the corresponding two cancer cell lines (HuH‐6, HepG2), human breast epithelial cell lines (MCF‐10A) and the corresponding two cancer cell lines (MDA‐MB‐231, HCC1937), and human lung epithelial cells (BEAS‐2B) and the corresponding cancer cell line (A549). Experiments were run per triplicate (*n*=300). ns: *p* > 0.5, ^*^: 0.01 ≤ *p* ≤ 0.05, ^**^: 0.001 ≤ *p* ≤ 0.01, ^***^: *p* < 0.001. b) Fitting plot of actual cell mixing ratios versus impedance‐based calculations. C/N is denoted as the cancer‐to‐noncancerous cell ratio and error bars represent 95% confidence intervals, *n* = 10 independent replicates. c) Impedance distinguishes map of mixed stained cell samples. d) Receiver operating characteristic (ROC) curve for assessing the sensitivity of the impedance‐based distinguishing method.

As shown in **Figure** **4**a, all the cancer cells exhibited higher average impedance magnitude compared to their noncancerous counterparts across the tested liver, breast, and lung cell lines, with significant statistical differences. This difference is primarily attributed to distinct physical characteristics and metabolic activities inherent to cancer cells.^[^ ^43^, ^44^ ^]^ This consistent increment of impedance provides a reliable method for distinguishing cancer cells from noncancerous cells based on single‐cell impedance magnitude measurements. For example, in breast cells, noncancerous breast cells (MCF‐10A) showed impedance magnitude ranging from 1.2 × 10^7^ to 2.6 × 10^7^ ohms, while breast cancer cells (including MDA‐MB‐231 and HCC1937 cell lines) exhibited impedance magnitude ranging from 2.1 × 10^7^ to 4.7 × 10^7^ ohms. Therefore, cells with impedance magnitude above the upper threshold of noncancerous cells are likely cancerous, whereas those below the lower limit of cancer cells are likely noncancerous. However, cells with impedance magnitude within the range of 2.1 × 10^7^–2.6 × 10^7^ ohms cannot be definitively classified, so data within this impedance magnitude range were excluded from the analysis.

To enhance the realism of biopsy sample simulations, we combined MDA‐MB‐231 and MCF‐10A in varying proportions, denoted as the cancer‐to‐noncancerous cell ratio (C/N). We evaluated the correlation of impedance‐based calculations with these predefined mixing ratios, ranging from 0.25 to 4, as depicted in Figure 4b. The coefficient of determination was 0.95015, indicating a strong correlation and highlighting the precision of our platform in identifying unknown samples. To further validate the accuracy of our impedance‐based differentiation technique, we utilized distinct fluorescent markers— green for MCF‐10A cells and red for MDA‐MB‐231 cells—for biochemical identification. This approach allowed us to classify cells on the IC‐ECIS chip as cancerous or noncancerous based on their impedance readings, with fluorescence imaging serving as a comparative tool. The impedance differentiation map for these fluorescently marked mixed cell samples is illustrated in Figure 4c. Classification accuracy was high for samples in the second and fourth quadrants but lower for those in the first and third quadrants. Overall, our method achieved an accuracy rate of 81% across 300 samples.

To evaluate the precision of our IC‐ECIS technique for cell classification, we employed receiver operating characteristic analysis. This method plots sensitivity against (1 – specificity), representing the true positive rate and false positive rate of cell identification, respectively. Points closer to the top‐left corner indicate higher predictive accuracy. For a detailed understanding of the ROC model, please refer to the Supporting Information. Figure 4d illustrates the ROC analysis results for breast, liver, and lung cancers. Notably, our IC‐ECIS‐based classification for these cancer types predominantly falls in the upper left quadrant, indicating a sensitivity above 70% and a false positive rate below 20%. This performance significantly surpasses random predictions. The data indicate that fewer than 30% of cancer cells were incorrectly identified as noncancerous, and less than 20% of non‐cancerous cells were falsely detected as cancerous. The misclassification was caused by cell heterogeneity. Variations within cancer cell populations in terms of size, morphology, and membrane characteristics can lead to considerable overlap in impedance.^[^ ^45^ ^]^ Cell cycle differences also introduce substantial variations in electrical impedance due to changes in cell volume and membrane capacitance during different cycle phases, such as G₁/S and G₂/M, potentially affecting classification accuracy.^[^ ^46^ ^]^ Other objective factors like residual electronic noise and inconsistencies in cell positioning can also obscure subtle impedance differences. Despite the errors, we can still filter out more than 80% of normal cells in the primary tumor compared with traditional drug screening.

### 2.4 Drug Screening of Commercialized Cell Lines on the IC‐ECIS Chip

The IC‐ECIS method distinguishes itself from traditional physical and chemical approaches^[^ ^47^, ^48^ ^]^ for single‐cell classification due to its non‐destructive, non‐invasive, and real‐time capabilities. This innovative method ensures the preservation of cell viability post‐classification, rendering cells suitable for subsequent drug efficacy analyses. To assess the feasibility of using the IC‐ECIS chip for drug screening, we employed MDA‐MB‐231 and MCF‐10A cell lines as models for breast cancer and noncancerous cells, respectively. Cell viability was evaluated by staining dead cells with ethidium homodimer‐1, which produces red fluorescence, enabling accurate estimation of viable cell counts. For comparison, off‐chip drug screening using cell counting kit‐8 was conducted in parallel.

**Figure 5:**
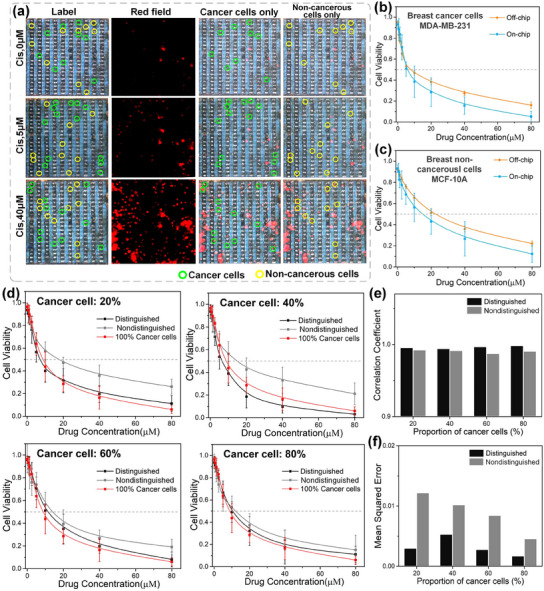
Drug screening of breast cancer cells and noncancerous breast cells. a) Image results for on‐chip results of 0, 5, and 40 µm Cis‐treated MDA‐MB‐231 cells and MCF‐10A cells for 24 h after labeling cell types. b,c) Drug sensitivity test results for Cis‐treated MDA‐MB‐231 cells and MCF‐10A cells on IC chip and off‐chip (96‐well plate). d) Drug screening curve of breast cancer without distinguishing and IC‐based distinguish at different cancer cell ratios. Error bars represent standard deviation, *n* = 3 independent replicates. e) The correlation coefficient of distinguished method and no distinguished method. f) Mean square error of distinguished method and no distinguished method.

In this study, we prepared a mixture of cancer and noncancerous cells in equal proportions (1:1 ratio) and deposited them onto an IC‐ECIS chip. Impedance measurements were conducted across all electrodes to distinguish between cancerous and noncancerous cells. As depicted in **Figure** **5**a, ≈50% of the identified cells were cancerous, consistent with the initial mixing ratio. To prevent disturbance to cell positioning from drug addition, we mixed cells with varying concentrations of the anticancer drug cisplatin (Cis)directly on the IC‐ECIS chip prior to experiments. These samples were then incubated for 24 h. Figure 5a illustrates a higher mortality rate among cells exposed to higher concentrations of Cis. Using the initial cell type identification, we separately analyzed the drug response of cancer and noncancerous cells. This approach enabled us to evaluate both the efficacy of the drug against cancer cells and its toxicity toward noncancerous cells simultaneously for the first time.

As shown in Figure 5b,c, cell viability decreased as drug concentrations increased for both cell lines. The half maximal inhibitory concentration (IC_50_) or cancer cells were 7.33 µm using the on‐chip method and 8.91 µm using the off‐chip method, confirming the IC‐ECIS chip's effectiveness in cancer drug screening. On‐chip, noncancerous cells showed a slightly lower IC_50_ value of 16.31 µm compared to 22.79 µm off‐chip, possibly due to their increased sensitivity to stress induced by the IC‐ECIS chip and electrical signals. Despite larger error bars in on‐chip measurements, likely due to lower cell numbers (hundreds on‐chip vs thousands off‐chip), the consistent trend of reduced cell viability with higher drug concentrations was observed with the IC‐ECIS chip. This consistency validates its utility for simultaneously identifying effective drugs and assessing drug toxicity using standardized cell lines in a single assay.

To simulate the situation of unknown tumor samples, we prepared a mixture of cancer and noncancerous cells in a list of proportions (2:8, 4:6, 6:4, and 8:2) and deposited them onto the IC‐ECIS chip. Analysis of the anti‐cancer drug efficacy with distinguished or no distinguished cells was run in parallel to demonstrate the advantages of our method over traditional methods without cell distinguishment. The drug screening results of 100% cancer cells were compared as the gold standard.

As shown in Figure 5d, cell viability decreased as drug concentrations increased in both cell proportions. The correlation coefficient presented in Figure 5e indicates that the curves of both distinguished and no distinguished groups exhibit a similar trend to the gold standard. However, the trend for the distinguished group aligns more closely with the gold standard. The mean square error (MSE) quantifies the accuracy of the numerical matching between the two curves. A smaller MSE value signifies that the curves are more closely aligned. As illustrated in Figure 5f, the drug screening curve of the distinguished group shows a stronger fit to the gold standard compared to that of the no distinguished group. As the proportion of cancer cells increases, the no distinguished group's curve gradually converges with the gold standard. It shows that under different cancer cell proportions, the accuracy of drug screening distinguished by IC‐ECIS is always higher than that of traditional drug screening methods without distinguishment.

### 2.5 Clinical Liver Cancer Cell Discrimination on the IC‐ECIS Chip

Tumors are categorized into benign and malignant types, with malignant tumors being cancerous and necessitating treatment, while benign tumors generally do not. Accurately classifying primary tumor cells is crucial for effective cancer diagnosis. To further validate the IC‐ECIS chips’ ability to classify primary tumor cells and support drug screening, we assessed cell types derived from clinical samples of hepatocellular carcinoma, a common liver cancer, using the chip. Results were compared with traditional tissue staining methods for cell differentiation. Lenvatinib (Len), a targeted therapeutic drug for liver cancer, was employed as a model drug for screening on the IC‐ECIS chips and for investigating clinical applications in precision medicine.

**Figure 6:**
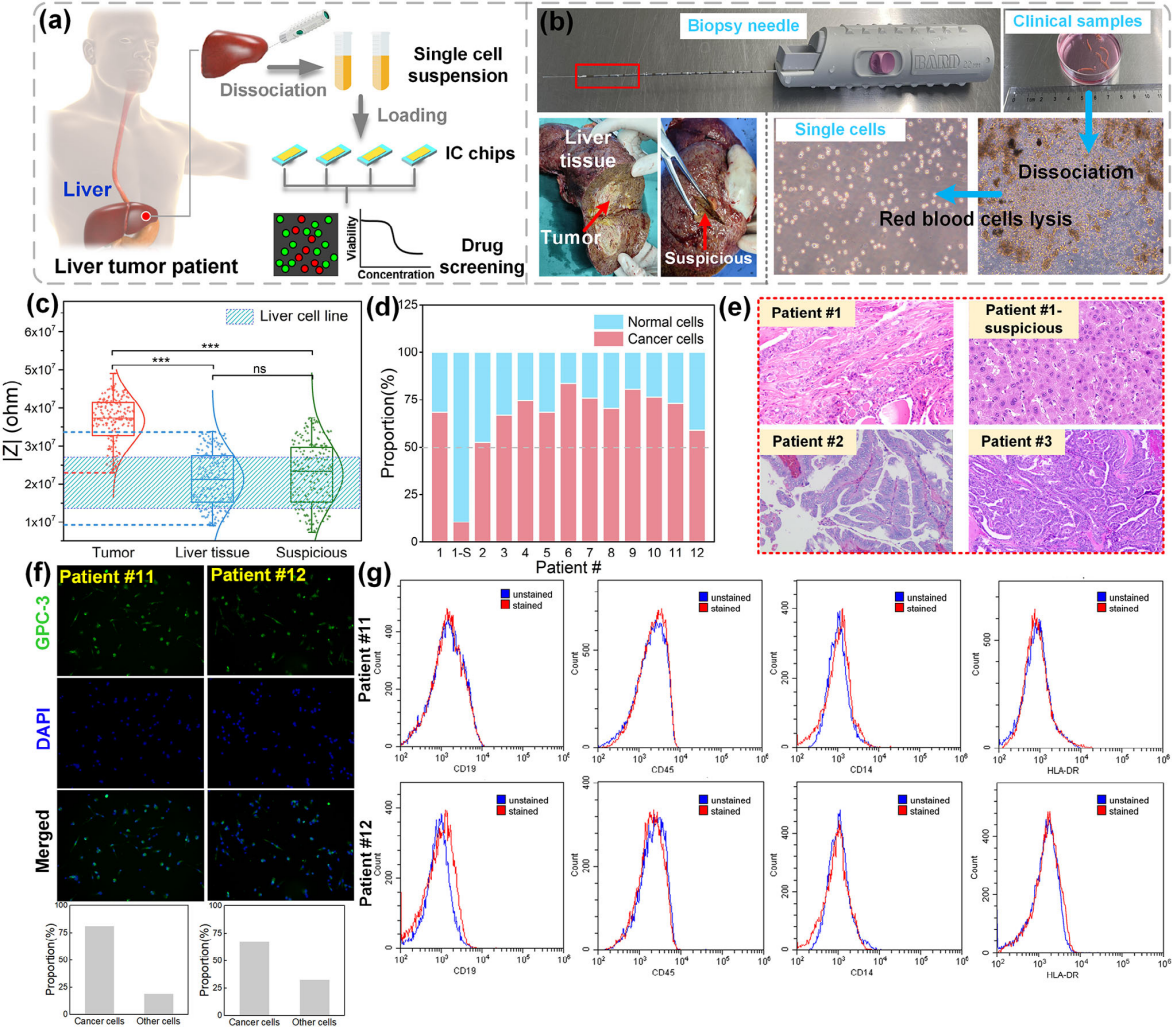
Clinical liver cancer cell discrimination and pathological verification. a) Schematic of drug susceptibility testing with HCC specimens on IC chips. b) Clinical sample biopsy sampling diagram. c) Impedance of tumor, liver tissue, and suspicious tissue from patient #1. Experiments were run per triplicate (*n* = 300). ns: *p* > 0.5, ^***^: *p* < 0.001. d) The proportion of cancer and noncancerous cells in the tumor tissue of different patients. e) Immunohistochemical staining pathological result of the liver tissue. f) Immunofluorescence results of tumors from patients #11 and #12. g) Flow cytometry analysis of proteins associated with B cells and T cells from patients #11 and #12.

**Figure** **6**a outlines the protocol for primary tumor assessment. The procedure involved dissecting liver cancer and using traditional staining techniques to detect cancer in clinical settings. For IC‐ECIS analysis, fresh samples of noncancerous liver tissue were obtained using biopsy needles, as depicted in Figure 6b, specifically from tumor‐free areas. Liver tumor specimens were extracted from various locations within the tumor mass. Additionally, tissues from suspected tumor sites were sampled to evaluate the IC‐ECIS method's capability to identify precancerous conditions. Cell types were identified with impedance analysis, and subsequently used to determine the drug efficacy on cancer cells and drug toxicity on noncancerous cells.

Figure 6c demonstrates impedance magnitude measurements taken from cells sourced from noncancerous liver tissue, tumor tissue, and tissue suspicious for tumor presence in Patient #1. Both theoretical analysis and experimental studies on various cell lines have indicated that cancer cells typically exhibit higher impedance magnitude compared to non‐cancerous cells, consistent with clinical findings. However, due to potential experimental variations and the sensitivity of the measurement system, there may be instances where impedance values overlap between cancerous and noncancerous cells. To accurately determine the percentage of cancer cells in a sample, we established a threshold based on the highest impedance value recorded for noncancerous cells in the patient's biopsy outside the tumor region. Cells with impedance magnitude below this threshold were classified as noncancerous, while those above it were identified as either cancer cells or cells in the process of undergoing carcinogenic transformation — targets for further investigation and elimination.

Based on the established criteria, we determined the proportions of cancer cells in tumor samples from patients #1 to #12, as depicted in Figure 6d. Our analysis revealed that a majority of these samples contained over 50% cancer cells, strongly indicating the tumor to be malignant, consistent with traditional clinical pathology findings. Notably, patient #2′s tumor sample showed a cancer‐to‐noncancerous cell ratio roughly 1:1, likely due to the small size of the biopsy obtained during liver resection, which posed challenges for accurate assessment. Further details and clinical images of the tumor are provided in the Supporting Information, highlighting that the small tissue sample included some non‐tumor cells as well. Additionally, a suspicious tissue sample from Patient #1 (referred to as Patient #1‐S) contained 11.5% cancer cells, below the typical 20% false positive rate observed in commercial cell lines, suggesting it could be a benign tumor.

Figure 6e displays pathology images of samples obtained from collaborating hospitals using traditional staining methods. These images clearly depict cancerous cells in samples from patients, aligning with our classification using the IC‐ECIS method. Sample #1‐S showed cellular characteristics resembling noncancerous liver tissue and was clinically diagnosed with inflammation, reinforcing the accuracy of our IC‐ECIS method for single‐cell discrimination in clinical settings. Besides, we also performed immunofluorescence experiments on tumors from patients #11 and #12 using the liver cancer‐specific antibody GPC‐3. The proportion of cancer cells obtained from IC‐ECIS was almost consistent with immunofluorescence results, as shown in Figure 6f. This consistency highlights the method's reliability and its potential application in clinical cancer diagnostics.

Tumor specimens collected from patients typically contain immune cells. To investigate the amount of potential immune cells remaining in the dissociated samples for IC‐ECIS detection, we performed a flow cytometric analysis on clinical tissues. Tumors were dissociated into single cell suspensions, and then the red blood cells in the cell suspension were lysed. We ran an analysis of CD34, CD45, CD14, CD19, and HLA‐DR, which are associated with Hematopoietic and immune cells.^[^ ^47^ ^]^ As shown in Figure 6g, the peaks were similar for all the markers and showed no difference for stained and unstained cells, indicating no many immune cells existed in the biopsy sample. Thus, it was reliable to distinguish cancer cells from noncancerous cells based on IC‐ECIS.

### 2.6 Drug Screening of Clinical Liver Cancer on the IC‐ECIS Chip

After cancer cells were distinguished from noncancerous cells in tumor tissues on the IC‐ECIS chip, a drug was administered to the chip, and the cells were cultured for 24 h for the purpose of drug screening. Given that liver cancer is widely recognized for its resistance to chemotherapy, Len, a drug that specifically targets genetic mutations in c‐KIT, FGFR1, FGFR2, FGFR3, FGFR4, PDGFR‐α, RET, FLT‐1, KDR, and FLT‐4,^[^ ^49^ ^]^ was selected as a model for this study. In clinical, Len showed an overall response rate of 24.1% for advanced hepatocellular carcinoma.^[^ ^50^ ^]^ It is anticipated that tumors harboring mutations in these genes will respond to treatment with Len. In this experiment, 10 µm of Len was applied as recommended by the pharmaceutical company.

Due to the distinct marking of cancer cells and noncancerous cells, it is possible to simultaneously analyze cell viability post‐treatment to evaluate the drug's efficacy in cancer cells and its toxicity to noncancerous cells. A 50% cell viability threshold was established to ascertain drug sensitivity in the cells. As indicated in **Table** **1**, cancer cells from patients #3, #5, and #8 were sensitive to Len, whereas only the noncancerous cells from patient #8 showed a response.

**Table 1:** On‐chip drug screening and sequencing results of clinical patients.

No.	On‐chip				Len related genes										Clinical data		
	cancer cells		noncancerous cells		c‐KIT	FGFR1	FGFR2	FGFR3	FGFR4	PDGFR‐α	RET	FLT‐1	KDR	FLT‐4	drug	AFP [ng mL^−1^]	
	cell viability with drug treatment (%)	drug efficacy	cell viability with drug treatment (%)	drug toxicity													
																Before drug treatment	After drug treatment
1	63.66	N	71.23	N	‐	‐	‐	‐	‐	‐	‐	‐	‐	‐	Sin	113.12	78.81
2	82.13	N	77.35	N	/	/	/	/	/	/	/	/	/	/	Len	66.18	64.52
3	43.71	Y	52.77	N	‐	‐	+	‐	‐	‐	‐	‐	‐	‐	N	/	/
4	76.67	N	83.46	N	‐	‐	‐	‐	‐	‐	‐	‐	‐	‐	Ate	51.39	37.66
5	37.21	Y	66.36	N	‐	‐	+	‐	‐	‐	+	‐	+	‐	N	/	/
6	69.38	N	76.13	N	‐	‐	‐	‐	‐	‐	‐	‐	‐	‐	N	/	/
7	82.43	N	86.55	N	‐	‐	‐	‐	‐	‐	‐	‐	‐	‐	Tis	101.72	73.61
8	41.23	Y	43.56	Y	‐	‐	‐	‐	‐	‐	‐	‐	‐	‐	N	/	/
9	79.15	N	64.33	N	/	/	/	/	/	/	/	/	/	/	N	/	/
10	89.15	N	81.38	N	/	/	/	/	/	/	/	/	/	/	N	/	/

Note: “+”, mutated; “‐”, no mutation; “/”, no data.

The remaining samples from the on‐chip drug screening were subsequently analyzed through sequencing to detect any mutations. No sequencing data were available for samples from patients #2, #9, and #10 due to the limited amount of clinical samples. According to Table 1, patient #3 exhibited a mutation in FGFR2; patient #5 had mutations in FGFR2, RET, and KDR, whereas no mutations were detected in these genes for patient #8.

FGFR2 encodes a fibroblast growth factor receptor. Statistically, 10%–15% of liver cancer patients exhibit gene fusion or rearrangement in FGFR2, leading to activation and transmission of genetic information to downstream pathways that promote tumor growth and angiogenesis,^[^ ^51^ ^]^ which explains the drug sensitivity observed in patient #3. In patient #5, mutations were found in RET and KDR as well. RET, a proto‐oncogene, encodes a transmembrane tyrosine protein kinase receptor that conveys signals for cell growth and differentiation.^[^ ^52^ ^]^ Mutations in RET can lead to uncontrolled activation, resulting in dysregulated cell proliferation, migration, and differentiation.^[^ ^53^ ^]^ KDR encodes the vascular endothelial growth factor receptor‐2 (VEGFR‐2), a crucial receptor in the signaling pathway for vascular growth factors, highly expressed in most tumors, promoting vascular endothelial cell division and proliferation, as well as tumor vascularization and growth.^[^ ^54^ ^]^

These mutations confirmed the findings of the on‐chip drug screening for patients #3 and #5. In patient #8, Len was lethal to both cancerous and noncancerous cells, as shown in Table 1, although no mutations were found in the genes related to Len. This was attributed to multiple interventional treatments prior to tumor resection described in his medical record. This phenomenon may be attributed to several factors, including off‐target effects, undetected genetic or epigenetic alterations, or technical considerations such as prior treatments affecting cell viability. Len is a multi‐kinase inhibitor targeting receptors. Even in the absence of detectable mutations in drug‐related genes, lenvatinib may influence normal cell functions through unintended targets. These off‐target effects might not be captured by standard genetic testing and require proteomic or kinase activity profiling for further validation.^[^ ^55^ ^]^ Furthermore, prior treatments may alter cellular metabolic states and lead to false‐positive toxicity signals in vitro.

The comparison of drug screening results with clinical treatment outcomes was conducted in a double‐blinded fashion. Decisions regarding which patient would receive a drug and which specific drug to prescribe were made by the physicians without knowing the drug screening results. This comparison was a retrospective analysis. The effectiveness of the drug was assessed based on the level of alpha‐fetoprotein (AFP), a liver cancer biomarker.^[^ ^56^ ^]^ As shown in Table 1, Len had been administered to patient #2 prior to tumor resection. The AFP level was 66.18 ng mL^−1^ before treatment and remained at 64.52 ng mL^−1^ post‐treatment. This observation clearly indicates that the tumor did not respond to the targeted drug, which is consistent with the on‐chip drug screening results. Although sequencing data were not obtained due to limited sample availability, clinical findings corroborated the drug screening outcomes. Besides, Patient #2 had metastatic liver cancer that had spread to the diaphragm. Despite the complexity associated with metastatic lesions, our IC‐ECIS platform successfully achieved cell identification and drug screening. It preliminarily demonstrates the applicability of the IC‐ECIS platform to metastatic cancer. All analyses above indicate that the IC‐ECIS chip for drug screening could serve as a cost‐effective alternative to whole gene sequencing for cancer precision medicine.

## 3 Discussion and Conclusion

Gene expression evaluations constitute the predominant segment of the precision medicine market.^[^ ^57^ ^]^ Analysis of individual patient genomic data informs the selection of suitable drug treatments. However, predicting treatment outcomes based on specific gene mutations is challenging due to complex drug pathways involving multiple genes. Studies indicate that ≈30% of patients undergoing targeted therapy based on gene sequencing do not achieve the expected results.^[^ ^58^ ^]^ An alternative approach involves drug screening at the cellular level using primary tumor cells, offering immediate insights into the tumor's response to specific drugs. Yet, primary tumor drug screening encounters a significant challenge: biopsy samples often consist of a mixture of cancer and noncancerous cells, which may result in inaccurate drug efficacy assessments.

This paper introduces the IC‐ECIS chip, a platform that distinguishes cancer cells from noncancerous cells at the single‐cell level through the integration of IC technology and electrical impedance sensing. The chip features hundreds of surface microelectrodes and impedance measurement circuits within a 1 mm × 2 mm area. The efficacy of the IC‐ECIS chip for cell distinction and drug screening has been validated using commercially available human breast cancer cells, liver cancer cells, lung cancer cells, and their respective noncancerous cell lines. It was also validated with clinical liver tumor samples, comparing on‐chip drug screening results with exon gene sequencing and clinical drug treatments, demonstrating the potential of the IC‐ECIS to replace costly gene sequencing in cancer precision medicine.

Compared with the gold standard in gene sequencing for precision medicine, IC‐ECIS offers several advantages. 1) The cell types are identified prior to drug screening, which not only validates the efficacy of specific drugs against cancer cells but also underscores their toxicity toward noncancerous cells. 2) Being a non‐invasive, non‐destructive technique, the identified cells remain suitable for cell viability tests treated with drugs. 3) Primary tumor cell drug screening enables the evaluation of both targeted and chemotherapy drugs, whereas gene sequencing is limited to targeted drugs. 4) The complete process, from the collection of primary tumor samples to cell identification and drug screening, can be finalized within 36 h. 5) Conventional drug screening in microplates often faces challenges due to the limited quantity of primary tumor cells. In contrast, the IC‐ECIS system accommodates hundreds of cells, thus fulfilling the screening requirements without necessitating further subcultures. This avoids the introduction of unintended mutations in progeny cells, leading to more precise drug screening outcomes. 6) IC‐ECIS functions as an automated system for reading and analyzing ultraweak bioelectric signals. It does not require professional bioinformatics analysts to derive drug screening results. Furthermore, the device is designed to be portable, allowing for immediate experiments with the freshest samples in animal facilities or hospitals. 7) Our design utilizes 0.35‐µm CMOS technology, which is adequate for our purposes. To reduce costs, we use the multi‐project wafer (MPW) method to share a six‐inch wafer for multiple designs, thereby sharing the cost of the mask and wafer. The net cost per chip was $30. Actually, after the layout is determined, the cost of the mask is fixed and will not change with the increase in production. Therefore, in mass production, the cost of the mask can be amortized over more chips, and the amortization cost of one chip will be reduced to less than $10. The biggest cost comes from post‐processing of FIB ($5000). But it's based on laboratory research. In the future, in mass production, photolithography can be even cheaper on a whole wafer. The post‐processing cost per chip will be less than $1. Encapsulation includes wire bonding and sealing, and the cost is about $20. In general, for laboratory research, the cost of one chip is $5000. For large‐scale processing, the cost of one chip can be reduced to less than $30.8) Our samples are processed cells, not human fluids. So the chip can be reused for lab research purposes after cleaning. Once in mass production, they can be disposable.

Like other new‐emerging technologies, the IC‐ECIS chip also has some limitations. 1) The chip's throughput (352 electrodes/chip) is not high. To solve it, we are developing a mass‐scale chip processing technology based on embedded silicon packaging. This technology is to etch multiple chip‐sized buried grooves on the wafer, bury the chips, and then perform photolithography and electrode batch patterning at the same time. 2) The low cell capture rate limits scalability for large clinical samples. To address it, we aim to introduce dielectrophoresis to move individual cells precisely to the center of the electrode.

3) The limited sample size may impact the findings' statistical confidence and restrict the conclusions' generalizability. With a small cohort, inter‐patient variability and outlier responses could disproportionately influence overall trends. We aim to expand the scope of our research by including additional clinical samples and evaluating a broader range of therapeutic drugs to further enhance the clinical relevance and applicability.

This research marks the initial endeavor to apply IC technology to the field of precision medicine. In the future, this technology might evolve into a composite sensing system capable of measuring various ultraweak biological signals and cellular environmental changes, such as acidity, temperature, impedance, and potential, on a single chip. It may be further integrated with artificial intelligence for automatic analysis and combined with microfluidics for recovering drug‐resistant cells for further analysis. By combining microfluidics, circuit computation, and sensing functions, the microsystem aims to achieve small‐scale, rapid, and efficient clinical analyses, representing a substantial advancement toward realizing a true lab‐on‐an‐IC chip.

## Conflict of Interest

The authors declare no conflict of interest.

## Supporting information



Supporting Information

Supporting Information

Supporting Information

## Data Availability

The data that support the findings of this study are available in the supplementary material of this article.
