# Supplementary material for: Identification and Drug Screening of Single Cells from Human Tumors on Semiconductor Chip for Cancer Precision Medicine
Source: Adv Sci (Weinh). 2025 Apr 24;12(28):2503131. doi: 10.1002/advs.202503131 (PMC12302552; doi:10.1002/advs.202503131)
Supplement: Supplementary file 1 — Supporting Information [file ADVS-12-2503131-s002.docx]

**Electronic Supporting Information for Publication**

**Identification and Drug Screening of Single Cells from Human Tumors on Semiconductor Chip for Cancer Precision Medicine**

Wenhao Hui ^1, 2, #^, Kameng Lei ^1, 2, #^, Yingying Liu ^1, 2^, Xinru Huang ^3^, Yunlong Zhong ^4^, Xiaojun Chen^1,5^, Mingji Wei ^6^, Jie Yan ^7, 8^, Ren Shen^1, 2^, Pui-In Mak ^1,2^, Rui Martins ^1,2,9^, Shuhong Yi ^3^*, Ping Wang ^4^*, Yanwei Jia ^1,2,10,^ *

^1^ State Key Laboratory of Analog and Mixed-Signal VLSI, Institute of Microelectronics, University of Macau, Macau

^2^ Faculty of Science and Technology, University of Macau, Macau

^3^ Liver Transplantation Center, The Third Affiliated Hospital, Sun Yat-Sen University, Guangzhou, China

^4^ Department of Hepatobiliary Surgery, The First Affiliated Hospital of Guangzhou Medical University, Guangzhou, China

^5^ Lingnan Normal University, Zhanjiang, China

^6^ Electrical and Information Engineering, Jiangsu University, Zhenjiang, China

^7^ Department of Physics, National University of Singapore, Singapore

^8^ Mechanobiology Institute, National University of Singapore, Singapore

^9^ On leave from Instituto Superior Tecnico, Universidade de Lisboa, Lisboa, Portugal

^10^ MoE Frontiers Science Center for Precision Oncology, University of Macau, Macau

* Corresponding authors: [wangping1219@126.com](mailto:wangping1219@126.com); [yishuhong@163.com](mailto:yishuhong@163.com); yanweijia@um.edu.mo

**Materials**

Acetone, ethanol, and IPA were purchased from Millipore (USA). Cis-diammineplatinum (II), EP, Y27632, dexamethasone, nicotinamide, insulin, hydrocortisone, penicillin/streptomycin, N-acetyl-l-cysteine, cholera toxin, and hyaluronidase were purchased from Sigma Aldrich (Oakville, USA). Fetal bovine serum (FBS), phosphate buffer solution (PBS), roswell park memorial institute (RPMI) 1640, trypsin-EDTA, Dulbecco’s Modified Eagle’s Medium (DMEM), collagenase II, Hank’s Balanced Salt Solution (HBSS), Earle's balanced salt solution (EBSS), DMEM/F12, Glutamax, HEPES, and 1:50 B27 were purchased from Gibco. EthD-1, RBC lysis buffer, Cell Tracker™ Red, and Cell Tracker™ Green were purchased from Invitrogen (USA). The Primary Breast Cancer Medium (PRS-BCM-2D) was purchased from Zhongke Precision Biomedical Technology Co., Ltd. The Thyroid Carcinoma Organoid Culture Medium (OCMHC11-M050) was purchased from Novoprotein Technology Co., Ltd. The 40-μm nylon cell strainer, Lenvatinib (Len), cisplatin, and dimethyl sulfoxide (DMSO) were purchased from Solarbio (Beijing, China). Forskolin and A8301 were purchased from Tocris (England). Recombinant human EGF, recombinant human FGF10, and recombinant human HGF were purchased from Peprotech (USA). The disposable biopsy needle mc1816 was purchased from Bard (USA). MDA-MB-231 cells, HepG2 cells, and MCF-10A cells were obtained from the Faculty of Health Sciences, University of Macau (Macau, China). HuH-6 cells, L-02 cells, and HCC1937 cells were purchased from Jin yuan Biotechnology (Shanghai, China). B lymphocyte SU-DHL-10, Human leukemic T lymphocytes, A549 and BEAS-2B were purchased by Shanghai Jin Biotechnology (Shanghai, China). RGD linear peptides was obtained from SelleckChem (Germany). The antibodies used in this study, including Anti-CD133 [RM1002] (ab278053), Anti-CD44 [BLR038F] (ab243894), Anti-CD90/Thy1 [EPR28145-53] (ab307736), Anti-EpCAM [EPR20532-225] (ab223582), Anti-CD34 [EPR373Y] (ab81289), Anti-CD45 [EPR322Y] (ab40763), Anti-CD14 [SP192] (ab183322), Anti-CD19 [EPR5906] (ab134114), Anti-HLA-DR [EPR3692] (ab92511), and Anti-Glypican 3 [EPR20569] (ab207080) were purchased from Abcam (Cambridge, UK).

**Cell culture**

The MDA-MB-231 cells, MCF-10A cells, HepG2 cells, HuH-6 cells, L-02 cells, A549, BEAS-2B, SU-DHL-10, T lymphocytes and HCC1937 cells were cultured in a humidified incubator (37 °C, 5% CO_2_). The culture medium for the MDA-MB-231 cells, HepG2 cells, and HuH-6 cells was Dulbecco’s Modified Eagle’s Medium (DMEM), supplemented with 10% FBS and 100 U/mL penicillin-streptomycin. The culture medium for HCC1937 cells and L-02 cells was RPMI, supplemented with 10% FBS and 100 U/mL penicillin-streptomycin. The medium for MCF-10A was DMEM, supplemented with 5% Horse Serum, 20 ng/mL EGF, 0.5 mg/mL Hydrocortisone, 100 ng/mL Cholera Toxin, 10 μg/mL insulin, and 100 U/mL penicillin-streptomycin. The cells were cultured every 2-3 days for each passage at 2 × 10^5^ cells per cm^2^. The culture medium for B cells and T cells was RPMI-1640 medium, supplemented with 10% FBS, 1% glutamine, 1% sodium pyruvate, and 1% P/S penicillin-streptomycin. When cell density reached 80%-90% confluence, subculturing was performed. Cells cultured in suspension were maintained by adding fresh complete medium to the culture flask, ensuring the cell density remained between 1 × 10^5^ and 1 × 10^6^ cells/mL. Before the experiments, the cells were dissociated and resuspended in a fresh medium under the action of trypsin. The suspended cells were centrifuged at 1200 rpm for 3 minutes. The supernatant medium was removed to obtain cells. Fresh culture medium was then added into the centrifuge tube for an expected cell concentration.

**IC design**

The IC was designed and simulated in Cadence (Cadence Design Systems, Inc, USA), then implemented with 0.35-μm commercial standard complementary metal-oxide-semiconductor (CMOS) process by ams-OSRAM AG (Premstaetten, Austria). The interior structure of the IC chip consists of semiconductor components, insulation layers, vias, four metal layers, etc. The detailed parameters of the chip can be seen in Table S2. The periphery circuit was designed by Altium Designer (Altium, Australia) and simulated by Multisim (National Instruments, USA). The IC chip was connected to the printed circuit board (PCB) with 14 gold wires of 1 mil (7 lines on each side). The contact pads were sealed with black epoxy resin to avoid external interference by Suziite (Shanghai, China). The focused ion beam (FIB) was processed by Ion Beam Technology (Beijing, China). The surface heat distribution of the chip during operation is measured using a microscopic infrared heat distribution test system.

**Table S1** Parameters of IC chip

| Technology | AMS 0.35 μm CMOS |
| --- | --- |
| Area | 2.16×0.92 mm^2^ |
| Sensing Electrode Area | 0.67×0.47 mm^2^ |
| Number of electrodes | 352 |
| TIA number | 16 |
| Power consumption | 6.9597 mW |
| Supply voltage | 3.3 V |
| Clock of digital | 30 kHz |

In our TIA, we placed a feedback capacitor (*C_F_*) of ~150 fF to ensure the stability of the TIA on the following basis. We implemented the amplifier as a two-stage amplifier with a unity gain-bandwidth (*f*_GBW_) of 60 MHz. The simulated input fringe capacitance (*C*_i_) is ~20 fF (assuming the source is from the adjacent electrode). And the minimum transimpedance gain (i.e., the feedback resistor) setting is 20 kΩ. Altogether, the required *C_F_* is:

$$C_{F}=\frac{1+\sqrt{1+8\pi R_{F}C_{i}f_{GBW}}}{4\pi R_{F}C_{i}f_{GBW}} S (1)$$

Hence, we can get *C*_F_ = 150 fF. Detailed schematic of the TIA and the components value are shown in Fig. S1.


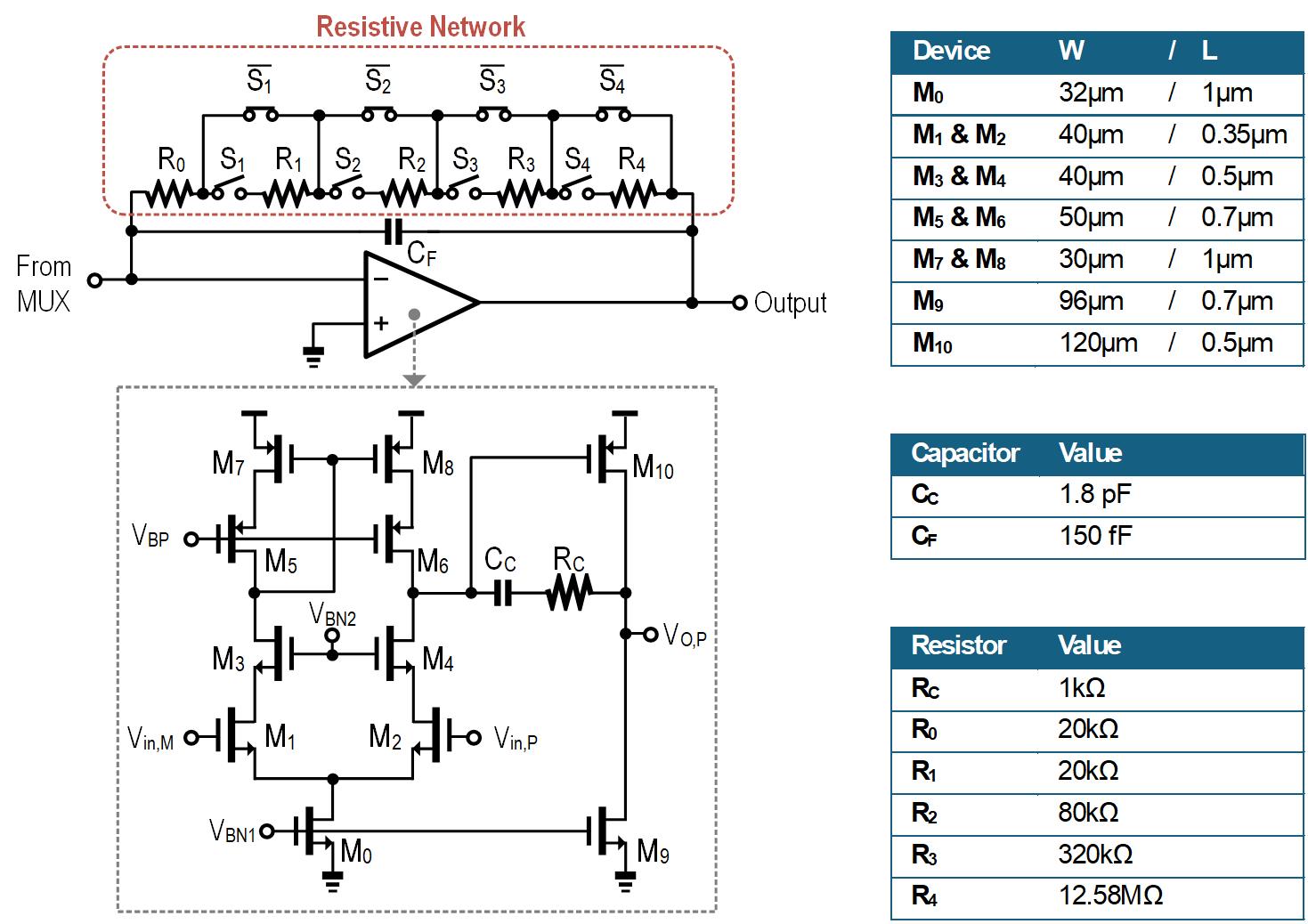


Fig. S1 Detailed schematic of the TIA and the components value.

**Fabrication process of the electrode array**

After exploring several micro-processing technologies, we ultimately chose the focused ion beam (FIB) with high processing precision, no mask, and no damage to the sample. We fabricated the chip in the AMS 0.35µm CMOS process. It has 4 metal layers and 2 polysilicon layers. The top metal has a thickness of ~925 nm, formed by 95% aluminum and 5% copper. The top metal layer does not have a passivation layer, ensuring a continuous metal surface across the chip, which facilitates the creation of the sensing electrodes. The electrodes were defined by ion etching after loading the design graphics of electrode array. We employed gallium ions with a probe current of 51 nA and an acceleration voltage of 30 kV to carve the electrode shape and etch away the metal between the electrodes. Gallium has a low melting point, low vapor pressure, and good oxidation resistance, making it an excellent choice^1^. Following that, we deposited a layer of biocompatible metal, platinum, to enhance cell adhesion to the electrode. The electrode shape is first drawn in CAD, then imported into the FIB device. Pre-experiments were imperative before etching the metal layer, and we adjusted the probe current and accelerating voltage appropriately to control the depth of the ion beam cutting and avoid damage to the underlying circuit.

**Optimization of surface electrode shape**

We have designed five shapes of sensing electrodes including square, interdigitated, circular, concentric, and conical electrodes. As shown in Fig S2, considering that the probes at the bottom of the electrode are a 10 µm × 20 µm square array, the concentric circle’s structure is not suitable for covering the probes on both sides. We further simulate the electrode field in CMOSOL. The electric field generated by the square electrode is weak, and the electric field strength distribution is relatively flat, lacking obvious concentrated areas. The electric field of the circular electrode is distributed in a bi-circular symmetric manner. The electric field is concentrated in the edge area of the electrode, but the intensity is uneven and there is an obvious central empty area. The concentric circle electrodes form a circular symmetrical electric field, and the electric field decays rapidly from the inner circle to the outside. It is suitable for single cell impedance measurement, but not suitable for our chip bottom probe structure. The electric field of the conical electrode is concentrated at the tip of the cone, which has high requirements for cell positioning. Compared with other electrodes, the interdigitated electrodes generate a highly concentrated electric field between the fingers, with rapid decay outside this region. Its electric field is concentrated between the two electrode fingers, which can effectively cover the scale of a single cell (about 10–20 µm), allowing single cells to be accurately sensed as they pass through. Besides, the electric field changes most dramatically in the interdigitated electrode area close to the electrode (<2 µm). This high electric field gradient allows small changes in cell impedance (such as cell size and membrane capacitance) to cause more significant electrical signal responses, which helps to improve the resolution of cell features.

**
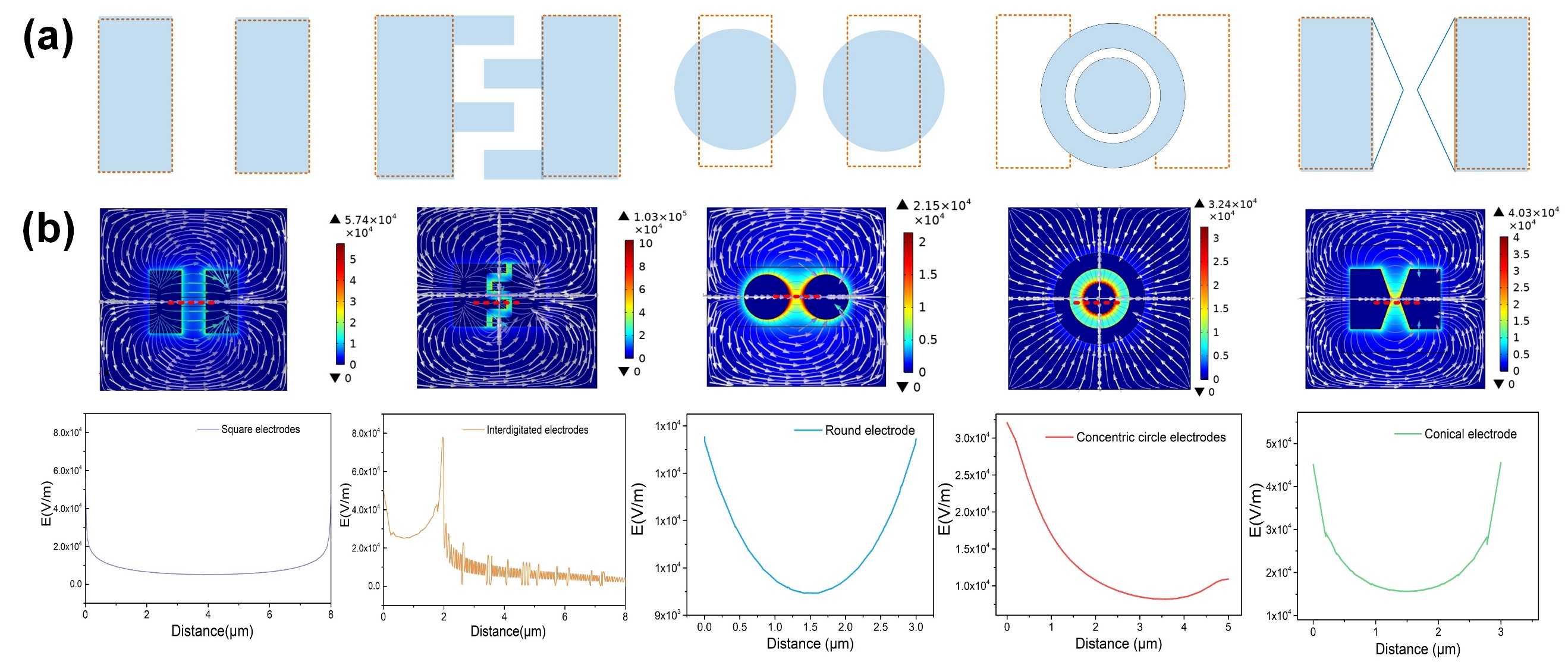
**

**Fig. S2** The design of different shape of sensing electrodes (a) and corresponding electric field distribution.

**System setup**

As can be seen from Fig. S3, the single-cell impedance sensing platform contained four parts: an IC chip and its peripheral circuits, software for calculating (MATLAB2018, USA), a signal generator (Tektronix, USA), and a microscope (Olympus, Japan). Cells were dissociated and resuspended in fresh culture media following standard procedures before the experiments. 2-5 μL cell suspension was transferred into the IC chip with a pipette (Eppendorf, Germany). We covered the liquid droplet with a 200 µm-thick acrylic film. Cells were deposited on the surface electrode under the action of gravity. Within 10–15 minutes, integrin clusters start recruiting cytoskeletal linker proteins (e.g., talin, vinculin), forming nascent focal adhesion complexes and providing initial cellular adhesion to the electrode surface. The impedance was measured when a single cell fell between the two electrodes. Multiple IC-ECIS chips are used in parallel for each patient sample to maximize the number of cells analyzed. The position of cells was determined under the microscope. The signal generator supplies sinusoidal excitation to the IC chip and the electrode channel at the corresponding position was opened. The impedance between the electrodes was recorded by the external circuit and forwarded to the field programmable gate array (FPGA) for processing. After each experiment, the chip was cleaned with isopropanol Alcohol (IPA), alcohol, and DI water and then dried with N_2_.

**
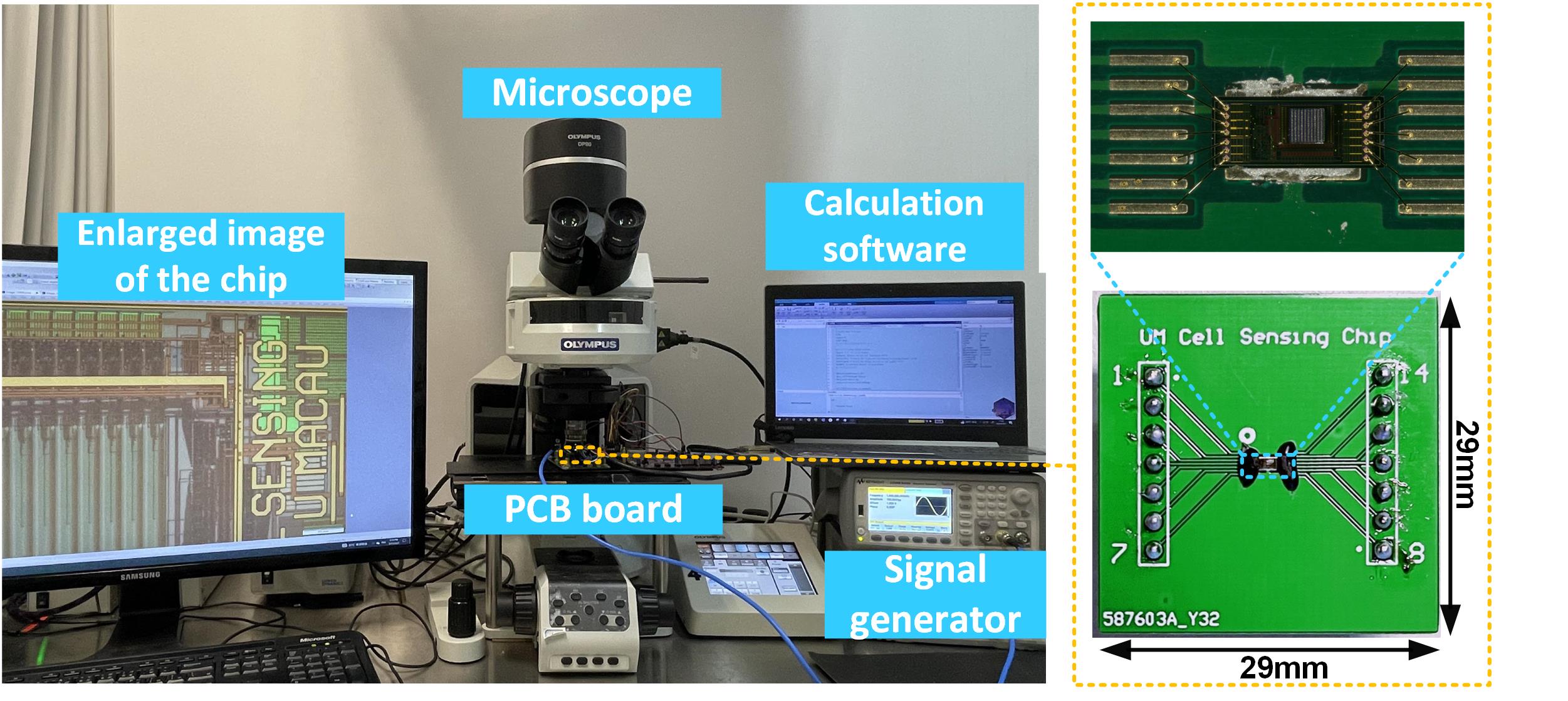
**

**Fig. S3.** The image of single-cell impedance sensing platform. It mainly includes four parts: an IC chip and its peripheral circuits, software for calculating, a signal generator, and a microscope. The sensing IC chip was connected to the peripheral circuit with gold wires.

**Equivalent circuit modeling**

*R_Ω_* denotes the solution resistance, indicative of the opposition to electric current flow within the electrolyte solution. In single-cell sensor, the value of *R_Ω_* can reflect the conductivity of the electrolyte solution within the sensor, with variations associated with factors such as the concentration and temperature of the electrolyte. Besides, *C_dl_*, the double layer capacitance, refers to the capacitive characteristic inherent to the double layer structure formed at the interface of an electrode and the electrolyte solution. *R_ct_*, known as the charge transfer resistance, signifies the resistive element encountered during the electrochemical reaction occurring at the electrode interface^39–42^. It is a pivotal parameter in gauging the rate of the electrochemical reaction, closely tied to the dynamics of the reaction kinetics. Thus, *R_ct_* can be utilized to monitor the kinetics of electrochemical reactions, such as the rate of antigen-antibody binding. Finally, *Z_W_*, the Warburg impedance, delineates the contribution of mass transport processes to the impedance in an electrochemical system, particularly within the low-frequency domain. *Z_W_* can reveal the diffusion behavior of antigens or antibodies on the sensor surface, as well as their binding status with the sensor interface, and the Warburg impedance is manifested as a straight line with a slope of 45° in graphical representations of impedance data. The impedance *Z_W_* is equivalent to the series connection of *R_W_* and *C_W_*, where *R_W_* = *σω*^-1/2^, *C_W_* = *σ*^-1^*ω*^-1/2^, *σ* is denoted as the constant of diffusion. *Z_W_* can be expressed as:

 $S (2)$

Thus, the total impedance *Z* is given by:

 $S (3)$

The real and imaginary part of impedance can be represented as:

 $S (4)$

When the frequency ω is lower than 1 Hz, the *Z_Re_* and *Z_Im_* can be simplified as:

 $S (5)$

The relationship of real and imaginary part can be expressed as:

 $S (6)$

Consequently, the diffusion-controlled representation on a Nyquist plot manifests as a straight line with a 45° inclination in the low-frequency and high-impedance regio. When the frequency ω is higher than 10 Hz, the term *ω*^-1/2^ can be disregarded.

 $S (7)$

The real and imaginary part of impedance can be represented as:

 $S (8)$

The relationship of real and imaginary part can be expressed as:

 $S (9)$

Consequently, the Nyquist plot is characterized by a semicircle centered at (*R_Ω_* + *R_ct_*/2, 0) with a radius of *R_ct_*/2 within the high-frequency domain.

**Finite element simulation**

The double-layer sphere model was introduced as a simplified approximation for the finite element simulation, to explore the general impact of cell size and position on the impedance signal. The finite element simulations follow Maxwell’s mixture theory. The inert nature of platinum specifically designates it as a polarized electrode, in that, at a particular DC bias charge can only accumulate at the electrode surface and polarize the electrodes. We must declare that the double-layer sphere model was introduced only as a simplified approximation for the finite element simulation, to explore the general impact of cell size and position on the impedance signal.

Throughout the simulation process, strict adherence is maintained to the following formulas.

|  | $\boldsymbol{E}=- \nabla\varphi,$ | $S(10)$ |
| --- | --- | --- |
|  | $\boldsymbol{J}=\left( \sigma+j\omega\varepsilon\right)\boldsymbol{E}=\sigma\boldsymbol{E}+j\omega\boldsymbol{D},$ | $S(11)$ |

where ***J*** (A/m^2^) represents the current density, ***E*** (V/m) denotes the electric field strength, $\varphi($V) represents the electric potential, $\sigma$(S/m) stands for conductivity, $j= \sqrt{-1}$, $\omega$ (rad/s) is the angular frequency, ***D*** (C/m^2^) is the electric displacement vector. The complex permittivity$\varepsilon^{'}$of cells is calculated using the following formula^37^:

|  | $\varepsilon'={\varepsilon'}_{m}\frac{2\left( 1-v_{in} \right){\varepsilon'}_{in}+\left( 1+2v_{in} \right){\varepsilon'}_{in}}{\left( 2+v_{in} \right){\varepsilon'}_{in}+\left( 1-v_{in} \right){\varepsilon'}_{in}},$ | $S(12)$ |
| --- | --- | --- |
|  | $v_{in}={(1-d/R)}^{3},$ | $S(13)$ |

where ${\varepsilon'}_{m}$ and ${\varepsilon'}_{in}$stand for the complex permittivity of the cell membrane and cell interior, respectively, *d* represents the thickness of the cell membrane, and *R* is the radius of the cell.

The finite element simulation parameters of noncancerous cell and cancer cell are indicated in Table. S2. Part of the simulation data comes from reference literature. Cell properties, such as conductivity, permittivity, and membrane thickness play an important role in electroporation.

**Table. S2** Finite element simulation parameters of noncancerous cell and cancer cell^2^.

|  | Noncancerous | Cancer |
| --- | --- | --- |
| **Conductivities (S/m):** |  | |
| Environment | 0.6 | 0.6 |
| Cell membrane | 5.6 × 10^-5^ | 9.1 × 10^-6^ |
| Cytoplasm | 1.31 | 0.48 |
| Nuclear envelope | 1.11 × 10^-2^ | 4.4 × 10^-3^ |
| **Relative Permittivity (F/m):** |  | |
| Environment | 80.0 | 80.0 |
| Cell membrane | 12.8 | 9.8 |
| Cytoplasm | 60.0 | 60.0 |
| Nuclear envelope | 106 | 60.3 |
| Nucleoplasm | 120.0 | 120.0 |
| Geometry parameters (μm): |  | |
| Radius of simulation region | 30.0 | 30.0 |
| Radius of cell | 3.3 | 5.2 |
| Thickness of cell membrane | 0.007 | 0.007 |
| Radius of nucleus | 2.8 | 4.4 |
| Thickness of nuclear envelope | 0.04 | 0.04 |

**Optimization of the cell density**

In addition, the number of available cells obtained from a single biopsy is limited for drug screening on primary tumors. It is necessary to improve single-cell capture efficiency to maximize the use of precious cells. Since the single cell capture between the electrodes was passive, the capture efficiency depended on cell concentration. Fig. S4a displays captured images of the MDA-MB-231 cells at concentrations of 10^5^, 5×10^5^, 10^6^, and 5×10^6^ cells/mL, respectively. On the array of 22 × 16 electrodes, image analysis software (ImageJ) was used to judge whether there are cells between two adjacent electrodes and generate a black-and-white diagram Fig. S4b. As can be seen, Fig. S4c shows the percentage of single cells trapped between electrodes at different cell concentrations. When the cell density increases from 10^5^ cells/mL to 10^6^ cells/mL, the efficiency increases from 7% to 15%. However, further increasing the cell density to 5×10^6^ cells/mL reduces the percentage of one cell between two electrodes. Consequently, we chose 10^6^ cells/mL as the optimal concentration for subsequent experiments.

**
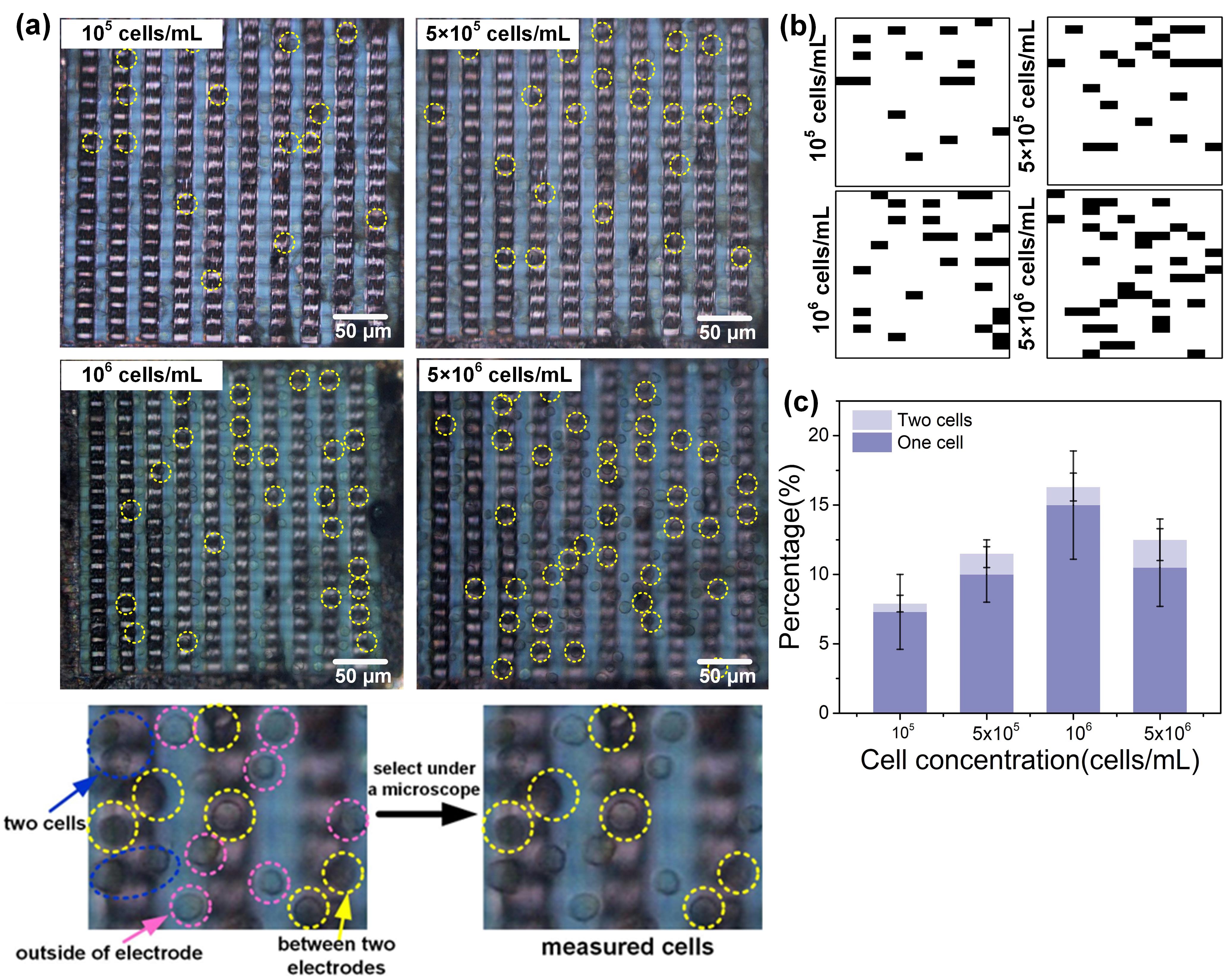
**

**Fig. S4.** Selection of optimal cell concentration. (a) Image of single cell captured on IC chip at 10^5^ cells/mL, 5×10^5^ cells/mL, 10^6^ cells/mL, and 5×10^6^ cells/mL. (b) Cell capture percentage in the black and white diagram. (c) The capture percentage of single cells and double cells under different cell concentrations.

To quantitatively analyze the effect of cell position on classification, we extracted the impedance for non-cancerous and cancerous cells falling on the electrode and for cells falling outside the electrode, as shown in Fig. S5. We found that the impedance change showed a consistent pattern with the simulation. The impedance of the cells outside the electrode was much lower than the impedance of the cells in the middle of the electrode.

**
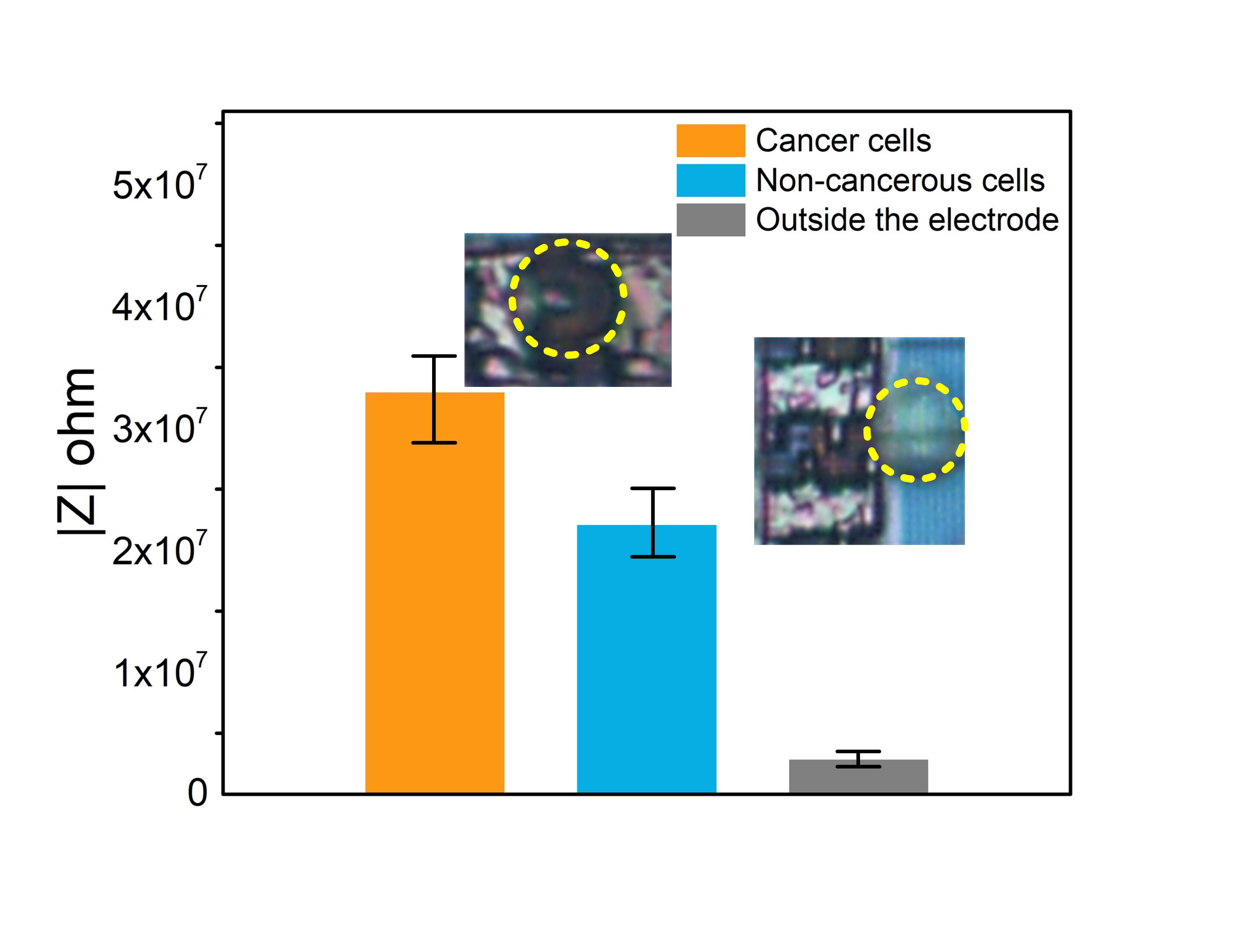
**

**Fig. S5** The influence of cell position changes on impedance measurement.

**Distinguish the cell mixture solution**

To simulate the natural conditions of biopsy samples, we mixed human breast epithelial cells (MCF-10A) and corresponding cancer cells (MDA-MB-231) in predefined ratios. The MDA-MB-231 cells and MCF-10A cells were digested with trypsin to make a cell suspension respectively. The suspended cells were centrifuged at 1200 rpm for 3 minutes. 5 μM Tracker™ Red was added into MDA-MB-231 cells suspension for staining and 5 μM Tracker™ Green was used for MCF-10A cells staining. After mixing for 10 minutes, centrifuge and discard the supernatant, then add the fresh culture medium into the centrifuge tube for a concentration of 10^6^ cells/mL. Finally, mix two cell suspensions in a series of ratios by setting the volumes of the two solutions. Utilize a pipette to add 2-5 μL of the mixed suspension onto the IC chips for impedance measurement.

**ROC curve**

To assess the sensitivity of our classifier, we introduced the receiver operating characteristic (ROC) line into the analysis of cells discrimination model^3,4^. The x-axis (1-specificity) represents the proportion of actual noncancerous cells that the test wrongly identified as cancer cells, while the y-axis (sensitivity) represents the proportion of actual cancer cells that the test correctly identified as cancer cells. The accuracy of this classifier model is shown in Eq. S (6).

|  | $ACC=\frac{TN+TP}{TN+FN+TP+FP} ,$ | $S( 14)$ |
| --- | --- | --- |

where the TN (True Negative) represents cells identified to be noncancerous and are noncancerous cells, FN (False Negative) stands for cells identified to be noncancerous cells but are cancer cells. TP (True Positive) represents cells identified to be cancer cells and are cancer cells. FP (False Positive) represents cells identified to be cancer cells but are noncancerous cells. The y-axis (sensitivity) of the ROC curve can be expressed as a true positive rate (TPR), which is shown in Eq. S (7):

|  | $TPR=\frac{TN}{TN+FP} .$ | $S( 15)$ |
| --- | --- | --- |

The x-axis (1-specificity) can be expressed as a false positive rate (FPR) which is shown in Eq. S(8):

|  | $FPR=\frac{FN}{FN+TP} .$ | $S( 16 )$ |
| --- | --- | --- |

According to the properties of the ROC curve, the closer the point to the upper left corner, the higher the prediction accuracy.

**Tumor microenvironments test**

To simulate real-world conditions of tumor microenvironments, a selection of cell types known for their relevance in tumor tissues was chosen for testing. This included the commercial human gastric epithelial cell line (GES-1), the corresponding cancer cell line (SGC-7901), as well as B lymphocytes, T lymphocytes, and mesenchymal stem cells (MSC), all of which may be present in tumor tissues.

As illustrated in Fig S6, cancer cells demonstrated a higher average impedance magnitude compared to normal cells, as well as B and T cells. MSC cells showed an impedance magnitude falling between normal cells and cancer cells. Conversely, T cells had a lower impedance magnitude than normal cells, while the B cells closely resembled normal cells in this regard. Previously reported findings attribute lower impedance profile on T-lymphocytes to their smaller size relative to other cell types, this aligns with our results. Statistical analysis confirmed a significant difference in the mean values between the normal and cancer cell populations, which can be attributed to the unique physical properties and metabolic activities of cancer cells. The IC-based impedance sensing serve as a reliable method for distinguishing cancer cells from non-cancerous cells based on single-cell impedance magnitude measurements, even in complex tumor microenvironments.


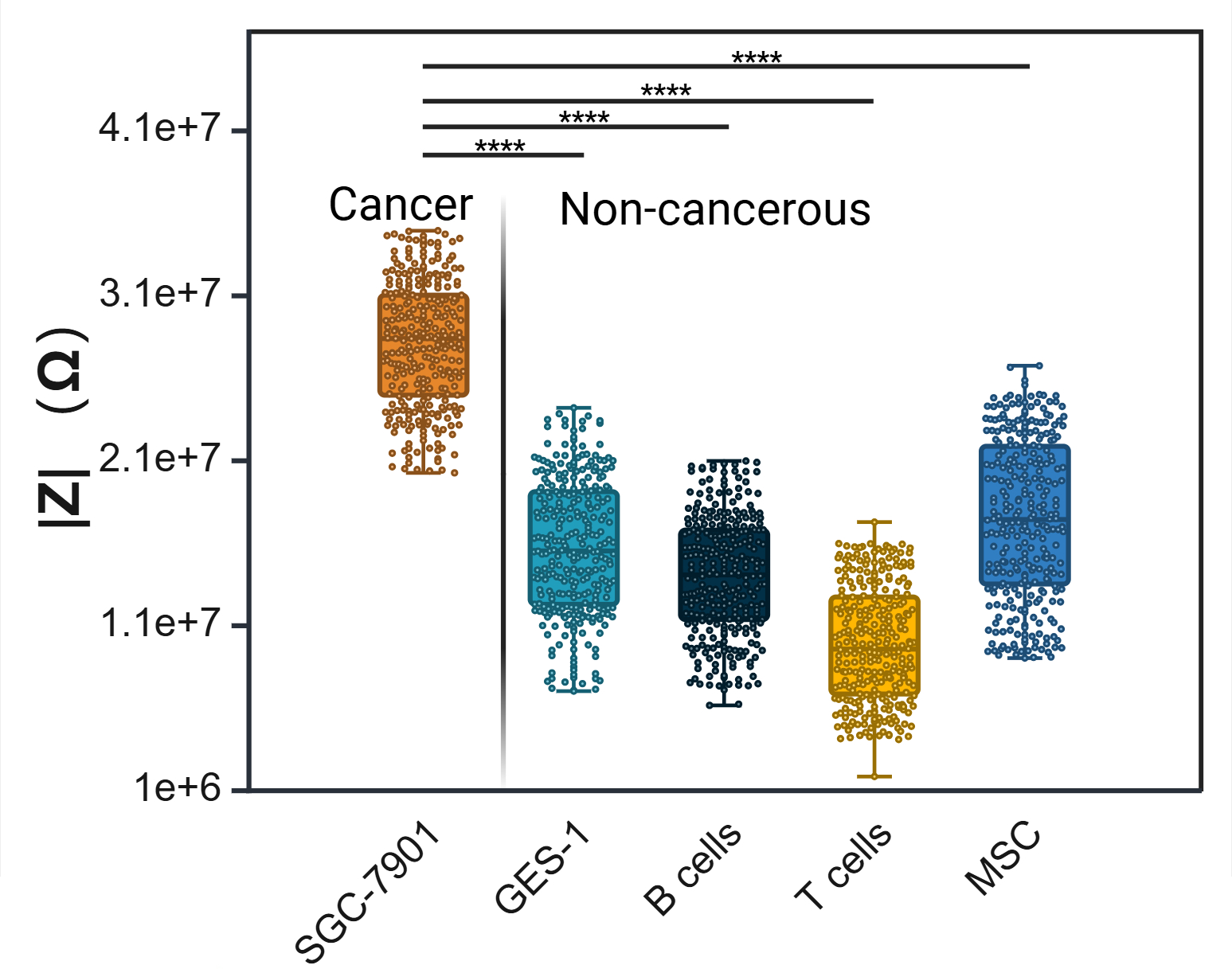


**Fig. S6** Impedance magnitude of human liver gastric epithelial cell line (GES-1), the corresponding cancer cell line (SGC-7901), B lymphocytes, T lymphocytes, and mesenchymal stem cells (MSC).

**Cell line drug screening on IC chip**

The chemotherapeutic reagent, cisplatin (Cis), was used in the drug sensitivity test. MDA-MB-231 breast cancer cells and MCF-10A noncancerous breast cells were used as the model cell lines. MDA-MB-231 cells and MCF-10A cells (10^6^ cells/mL) were combined with 2 μM EthD-1 and drug with a series of concentrations (0, 0.625, 1.25, 2.5, 5, 10, 20, 40, 80 μM). The mixed solution was loaded onto the IC chip, which distinguishes noncancerous cells from cancer cells based on impedance. Then, the chips were put in a cell culture dish containing wet paper towels and placed in a humidified incubator (37 °C, 5% CO_2_). After the co-culture of cells and drugs for 24 h, we evaluated the effects of the drugs by observing the cells under fluorescence microscopy. The fluorescent dye EthD-1 was used to identify dead cells, as it penetrates and stains cells that have lost membrane integrity. By analyzing the number of cells dyed, we could quantitatively assess cell viability and determine the effectiveness of the drug treatments. Since each cell has already been marked as noncancerous or cancerous, we can separately calculate the cancer cell viability and noncancerous cell viability, for cancer drug efficacy and toxicity simultaneously. The whole process is slightly longer than 24 hours. For clinical samples, another 3-5 hours of digestion was needed to obtain suspended cells before loading on chips for impedance measurement. The whole process was roughly 30 hours. If consider the sample delivery time, 36 hours was enough from sample to results.

**Drug screening off-chip**

The half-effective inhibitory concentration (IC_50_) of Cis for both MDA-MB-231 cells and MCF-10A cells was determined using a cell counting kit (CCK-8) assay. In the 96-well plate, 1.0 × 10^4^ cells were seeded in 100 μL of their respective cell culture medium per well. Considering the cells are isolated from each other in on-chip and off-chip experiments, the discrepancy in cell density would not affect the drug screening results. After adhering to the bottom of the wells, the cells in different wells were treated with various concentrations of Cis (with 0.1% dimethyl sulfoxide (DMSO) treatment as a negative control and a cell culture medium without cells as a background control) for 24 h. 4 mM Cis stock solution (dissolved in 50% DMSO solution) was used as the stock drug solution to generate a series of Cis concentrations. Then, 10 μL CCK-8 solution was added to each well and incubated for 0.5 h. All experiments were performed in triplicate. Finally, a microplate reader was employed to measure the absorbance at 450 nm (absorption wavelength of CCK-8). The absorbance readings were adjusted by subtracting the blank and then normalized against control wells.

**Impedance discrimination of HCC samples**

Human-derived cell lines were utilized as the most widely employed models for cancer treatments and biology studies. The experiments above were all based on commercial cell lines. Considering the clinical relevance of cell line models and clinical tumor samples from patients and the fragility of tumors^5–7^, we further evaluated the feasibility of impedance characterization and drug screening of clinical tumor samples on IC chips. The tumor patients’ clinical information is presented in Table S3 and photo of clinical tumor is shown in Fig.S7. The clinical conditions and surgical methods of different patients also vary.

More essentially, in some clinical situations, it is not just distinguishing specific cancer cells from the corresponding noncancerous tissue cells, such as liver cancer cells and liver tissue cells. The existence of metastatic cancer requires us to distinguish diaphragm epithelial cells from liver cancer cells, hepatocytes from cholangiocarcinoma cells, etc. In addition, various factors like the tumor size removed and the cancer stage will also affect the result. Therefore, clinical data is of great significance for verifying the impedance sensing method based on IC chips.

**Table S3** Tumor patients’ clinical information

| Patient  # | Age | Metastatic | Cancer type | Tissue  surrounding tumor | Surgery type |
| --- | --- | --- | --- | --- | --- |
| 1 | 53 | N | Liver cancer | Liver tissue | Liver transplantation |
| 2 | 55 | Y | Liver cancer | Diaphragm epithelial tissue | Tumor removal |
| 3 | 46 | N | Liver cancer | Liver tissue | Liver transplantation |
| 4 | 53 | N | Liver cancer | Liver tissue | Tumor removal |
| 5 | 62 | N | Liver cancer | Liver tissue | Liver transplantation |
| 6 | 53 | N | Liver cancer | Liver tissue | Tumor removal |
| 7 | 61 | N | Liver cancer | Liver tissue | Tumor removal |
| 8 | 57 | N | Liver cancer | Liver tissue | Tumor removal |
| 9 | 63 | N | Liver cancer | Liver tissue | Tumor removal |
| 10 | 49 | N | Liver cancer | Liver tissue | Tumor removal |
| 11 | 32 | N | Liver cancer | Liver tissue | Tumor removal |
| 12 | 69 | N | Liver cancer | Liver tissue | Tumor removal |


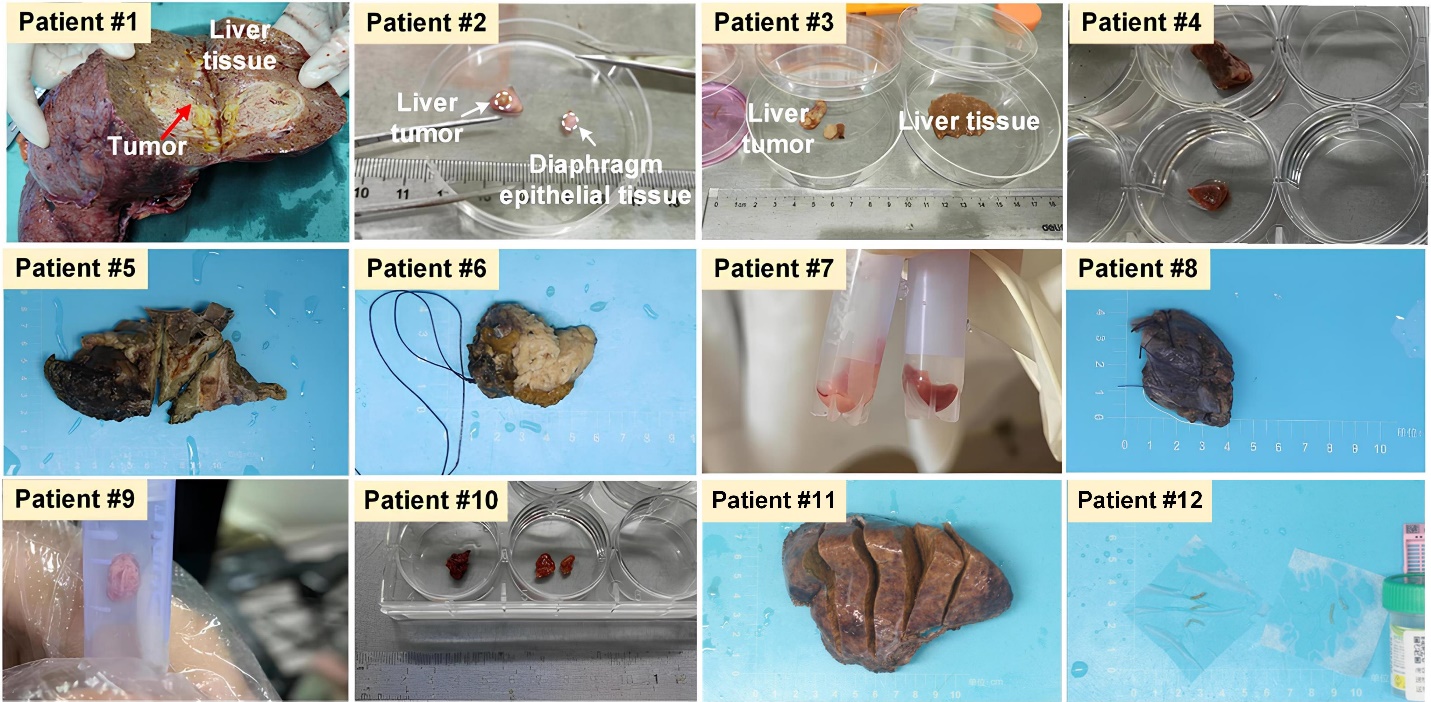


**Fig. S7.** Photo of clinical liver tumor.

**Drug screening with HCC specimens**

The protocol used in this study was approved by the University of Macau’s Research Ethics Board (Protocol # BRSERE23-APP009-IME). All the patients involved in this work have signed the consent forms before surgery. Tumor and surrounding tissues were obtained post-surgical hepatectomy. The clinical samples are from the Third Affiliated Hospital of Sun Yat-Sen University. The primary liver cancer culture medium is comprised of DMEM/F12, 1% penicillin/streptomycin, 10 mM nicotinamide, 50 ng/ml recombinant human EGF, 100 ng/ml recombinant human FGF10, 25 ng/ml recombinant human HGF, 1% Glutamax, 10 mM HEPES, 1:50 B27, 1.25 mM N-acetyl-l-cysteine, 10 μM forskolin, 5 μM A8301, 10 μM Y27632, and 3 nM dexamethasone. Immediately after surgery, clinical specimens were obtained from the excised liver tissue using a puncture biopsy needle 3 to 6 times and then transferred to the 15-ml centrifuge tubes with 5 ml of preservation solution (DMEM/F12 plus 1% penicillin/streptomycin, 1% Glutamax, and 10 mM HEPES). Each biopsy yielded tissue measuring 0.1 cm × 2 cm. The strip tissues were incubated at 37 °C with EBSS (supplemented with 125 U/ml collagenase Ⅱ and 0.1 mg/ml DNase I) in a culture dish for 3-6 h. The extent of digestion was observed, and the mixture was agitated using a pipette every half an hour to accelerate the digestion process until no large pieces of residue remained. After digestion, the cold DMEM/F12 was added to halt the process, and the mixture was filtered through a 40-μm nylon cell strainer (Solarbio) to get the single-cell suspension. The filtered mixture was transferred into a 50-ml tube after filtration and spun for 5 min at 300 × g. Subsequently, cells were resuspended in red blood cell lysis buffer (Invitrogen™) for 2 minutes, and enough EBSS was added to stop lysis. Given the complexity of clinical scenarios, intraoperative tumor specimens vary significantly in size and cell number. Actually, even the needle biopsy sample, commonly yield 10,000 or more single cells as shown in Fig. S8. With this scale, a 15% capture efficiency would still allow experiments to be conducted on over a thousand individual cells, a quantity sufficient to achieve statistically meaningful results for cell identification and subsequent drug screening analyses.

**
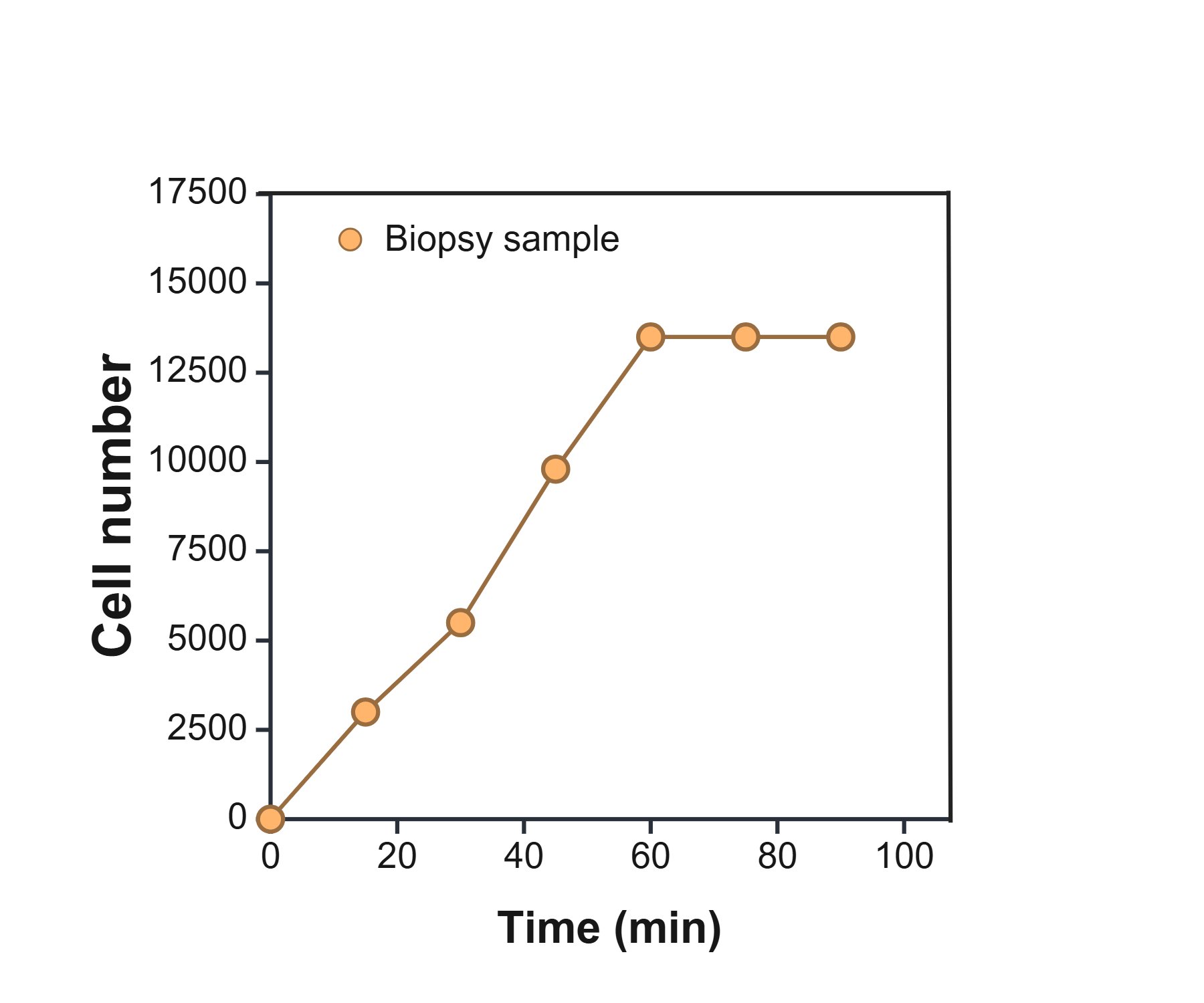
**

**Fig. S8** The number of single cells dissociated from biopsy sample.

The cells were then stained with Cell Tracker™ Green CMFDA Dye (Invitrogen™). The 10 μM Len and 0 μM-DMSO control were prepared and individually mixed with EthD-1. The concentration of EthD-1 after mixing was 2 μM. Cell droplets were loaded onto IC chips for impedance measurement. Then put IC chips in the incubator for the 24-hour drug efficacy test. The drug efficiency test was repeated thrice. We observed and recorded fluorescent cells under the fluorescence microscope and calculated cell viability after treatment with drugs. Cell Tracker™ Green Dye entered live cells and emitted green fluorescence. EthD-1 entered dead cells and emitted red fluorescence. Absolute cell viability was calculated as the number of green cells divided by the total number of both green and red cells. The relative cell viability under various drug treatments was normalized to the cell viability without the addition of drugs.

**Flow cytometry of tumor cells**

Freshly collected tissues were washed three times with PBS to remove debris. The tissues were then minced and digested in 0.1% collagenase at 37°C for 1-3 hours, with intermittent gentle vortexing 1-2 times to aid in tissue dissociation. Fresh complete medium was then added for continued cell growth. The attached cells were washed with PBS to remove any residual medium. The cells were then incubated with 1 mL of trypsin for dissociation. Once cells were detached, 2 mL of complete medium was added to neutralize the trypsin, and the cell suspension was collected. The cells were centrifuged at 1000 rpm for 5 minutes, the supernatant was discarded, and the cell pellet was resuspended in PBS. The cells were washed two more times with PBS, then resuspended in 500 μL of PBS with 0.1% BSA for further processing. To stain the cells, 1 μL of antibody was added to the cell suspension. The cells were gently mixed and incubated in the dark at room temperature for 5-10 minutes to allow for sufficient antibody binding. After incubation, the cells were washed to remove unbound antibody, and flow cytometry analysis was performed within one hour to ensure optimal signal detection and minimize cell degradation. The results were acquired using a flow cytometer equipped with appropriate lasers and filters. Data analysis was conducted using flow cytometry software (ST16 from Thermo Fisher company).

**Immunofluorescence of tumor cells**

Immunofluorescence staining was performed to detect specific markers in the tissue samples. Freshly collected tissues were washed three times with PBS, then minced and digested with collagenase at 37°C for 1-3 hours in a water bath. The dead cells were removed by washing with PBS, , and fresh medium was added to continue cell growth. After cells reached confluency, they were fixed directly on the culture dish using 500 μL of freshly prepared fixation buffer (4% paraformaldehyde) for 20 minutes at room temperature. Following fixation, the cells were washed three times with PBS, with each wash lasting 5 minutes to thoroughly remove the fixative. The fixed cells were incubated with a permeabilization buffer (0.1% Triton X-100 in PBS) for 10 minutes to allow antibody penetration. To prevent non-specific antibody binding, the cells were blocked with 3% BSA (bovine serum albumin) in PBS for 30 minutes at room temperature. After blocking, the cells were incubated with the primary antibody diluted in 3% BSA overnight at 4°C. The antibody was gently applied to ensure even distribution across the cell monolayer. The next day, the cells were washed three times with PBS to remove excess primary antibody. Then, the cells were incubated with the appropriate fluorophore-conjugated secondary antibody (diluted in 3% BSA) at room temperature for 50 minutes in the dark to protect the fluorophores from photobleaching. Following secondary antibody incubation, the cells were washed with PBS three times. To stain the nuclei, DAPI (4′,6-diamidino-2-phenylindole) was added for 5 minutes at room temperature. Excess DAPI was removed by washing the cells three times with PBS.

After the final wash, the coverslip was mounted on glass slides using an anti-fade mounting medium to preserve fluorescence. Fluorescence images were captured using a fluorescence microscope equipped with appropriate filter sets. The following wavelengths were used for visualization: DAPI (nuclear stain): excitation at 330–380 nm, emission at 420 nm. FITC: excitation at 465–495 nm, emission at 515–555 nm. CY3: excitation at 510–560 nm, emission at 590 nm. Fluorescence images were acquired and processed using image analysis software.

**Gene sequencing**

The genomic DNA was extracted from the tissues embedded in wax blocks. Patients #1, #3, #4, #5, #6, #7 and #8 underwent gene screening for variant screening and identification at Lingen Biotechnology Co., Ltd. (Shanghai, China). The total DNA was extracted using a standard DNA extraction protocol. Then the DNA which was fragmented by using sonication was subjected to library construction. Exome capture was performed using SureSelect Human All Exon V6 Kit (Agilent Technologies) following the vendor's recommended protocol. The sequencing was performed using the Illumina Novaseq™ 6000 with 150-bp paired-end sequencing mode. Millions of paired-end reads of 150bp in length were generated. Prior to alignment, the low-quality reads (1. reads containing sequencing adaptors; 2. nucleotide with q quality score lower than 20) were removed. Each base of each read has an associated quality score, corresponding to the probability of a sequencing error. Due to the Systematic biases, the reported quality scores are known to be inaccurate and as such must be recalibrated prior to genotyping. After recalibration, the recalibrated quality score in the output BAM will more closely correspond to the probability of a sequencing error. Variant calls can be generated with GATK HaplotypeCaller or UnifiedGenotyper, which Examines the evidence for variation from reference via Bayesian inference. A Gaussian mixture model is fit to assign an accurate confidence score to each putative mutation call and evaluate new potential variants. Biological functional annotation is a crucial step in finding the links between genetic variation and disease. Variant Effect Predictor (VEP) is utilized to add biological information to a set of variants.

**References:**

1. Shentu, J. *et al.* Characteristics for gallium-based liquid alloys of low melting temperature. *Metals (Basel)* **13**, 615 (2023).

2. Ermolina, L., Polevaya, Y., Feldman, Y., Ginzburg, B.-Z. & Schlesinger, M. *Study of Normal and Malignant White Blood Cells by Time Domain Dielectric Spectroscopy*. *1EEE Transactions on Dielectrics and Electrical Insulation* vol. 8 (2001).

3. Nahm, F. S. Receiver operating characteristic curve: overview and practical use for clinicians. *Korean J Anesthesiol* **75**, 25 (2022).

4. Polo, T. C. F. & Miot, H. A. Use of ROC curves in clinical and experimental studies. *Jornal vascular brasileiro* vol. 19 e20200186 Preprint at (2020).

5. Foo, M. A. *et al.* Clinical translation of patient-derived tumour organoids-bottlenecks and strategies. *Biomark Res* **10**, 10 (2022).

6. Lin, D. *et al.* Circulating tumor cells: biology and clinical significance. *Signal Transduct Target Ther* **6**, 404 (2021).

7. Sajjad, H. *et al.* Cancer models in preclinical research: A chronicle review of advancement in effective cancer research. *Animal Model Exp Med* **4**, 87–103 (2021).
